# Unpredictable Dynamic Behaviour of Ruthenium Chelate Pyrrole Derivatives

**DOI:** 10.3390/molecules29133068

**Published:** 2024-06-27

**Authors:** Giacomo Drius, Riccardo Tarroni, Matteo Birchmeier, Carola Parolin, Carla Boga, Magda Monari, Silvia Bordoni

**Affiliations:** 1 Department of Industrial Chemistry ‘Toso Montanari’, Alma Mater Studiorum, Università di Bologna, Via Piero Gobetti, 85, 40129 Bologna, Italy; giacomo.drius2@unibo.it (G.D.); matteo.birchmeier@studio.unibo.it (M.B.); carla.boga@unibo.it (C.B.); 2 Department of Pharmacy and Biotechnology, University of Bologna, 40127 Bologna, Italy; carola.parolin@unibo.it; 3 Department of Chemistry ‘Giacomo Ciamician’, Alma Mater Studiorum, Università di Bologna, Via Selmi 2, 40126 Bologna, Italy; magda.monari@unibo.it; 4 Health Sciences and Technologies Interdepartmental Centre for Industrial Research (CIRI SDV), University of Bologna, 40126 Bologna, Italy

**Keywords:** ruthenium, antibacterial, chelation change, isomerism, DFT, SCXRD

## Abstract

Reaction of [Ru(H)_2_(CO)(PPh_3_)_3_] **1** with an equimolar amount of pyrrole-2-carboxylic acid (**H_2_L_1_**) leads to the homoleptic chelate derivative k^2^(O,O)-[RuH(CO)(HL_1_)(PPh_3_)_2_] **2**. Prolonged acetonitrile refluxing promotes an unusual k^2^(O,O)- → k^2^(N,O)- dynamic chelate conversion, forming a neutral, stable, air- and moisture- insensitive, solvento-species k^2^(N,O)-[Ru(MeCN)(CO)(L_1_)(PPh_3_)_2_] **3**. Analogously, reaction of **1** with the pyrrole-2-carboxyaldehyde (**HL_2_**) affords k^2^(N,O)-[RuH(CO)(HL_2_)(PPh_3_)_2_] **4**, **5**, as a couple of functional isomers. Optimized reaction conditions such as temperature and solvent polarity allow the isolation of dominant configurations. Structure **5** is a pyrrolide Ru-carbaldehyde, obtained from cyclization of the pendant CHO function, whereas species **4** can be viewed as an ethanoyl-conjugated Ru-pyrrole. Derivatives **3**–**5** were characterized by single crystal X-ray diffraction, ESI-Ms, IR, and NMR spectroscopy, indicating distinct features for the Ru-bonded pyrrolyl groups. DFT computational results, coplanarity, bond equalization, and electron delocalization along the fused five-membered rings support aromatic features. In accordance with the antisymbiotic *trans*-influence, both the isolated isomers **4** and **5** disclose CO ligands opposite to N- or O-anionic groups. The quantitative Mayer bond order evidences a stabilizing backbonding effect. Antibacterial and antifungal trials on Gram-positive (*Staphylococcus aureus*), Gram-negative (*Escherichia coli*), and Candida albicans were further carried out.

## 1. Introduction

**Figure 1:**
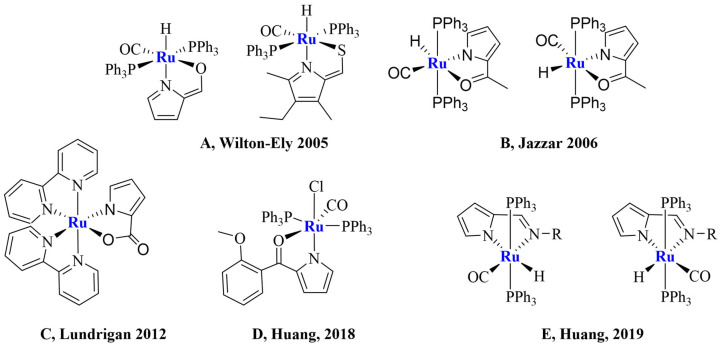
Selected examples of the previously reported Ru(II)-pyrrolyl complexes [21,22,23,24,25].

In the last decades, extensive research on Ru(II) pyrrolyl complexes bearing keto or ester functions has been conducted [1,2,3,4,5,6,7,8,9]. Part of their success is ascribed to their bioactivity as pharmacophores in various polycyclic heme-like metal systems. Azaheterocycle motifs are commonly present in the majority of biochemical processes and constitute the structural scaffolds for most of the commercially available pharmaceuticals and targeted issues in medicinal chemistry. Along this class of compounds, pyrroles are the five-membered aromatics most widely used in the pharmaceutical industry as anticancer, anti-inflammatory, antibacterial, antiviral, antihypertensive, and enzyme-modulating agents [9,10,11,12,13]. A promising biological activity shown by several complexes suggests the pivotal role of metals in enhancing the mechanical stability to shuttle bioactive fragments as intact to the targeted tissue. This action may prevent earlier degradation, limiting adverse side reactions, and pursuing therapeutic goals with reduced doses by hastening benign biological responses [14,15]. In pharmaceutical chemistry, where organic drugs have traditionally held prominence, metal complexes have become increasingly popular as diagnostic tools as well as anticancer or antibacterial agents. As a result of their ability to bind biomolecules, ligand exchange kinetics and iron-mimicking properties are anticipated to emerge as a novel metal-based drug generation [16,17]. Hence, the combination of pyrrole derivatives with Ruthenium might provide valuable insights into the functions of these hybrid species. Pyrrole derivatives (with O-, N-, or S- functionalized side arms) are well-known for a variety of catalytic and biochemical applications [4,5,6,7,8,9,10,18], and their metal coordination has been extensively explored on complexes showing various co-ligands [11,12,13,19,20]. In this context, it is worth citing the pioneering work of the Wilton–Ely group (A, Figure 1), leading to mixed-(N,X) chelate pyrrole-2-carboxaldehyde (X = O) and carbothioaldehyde (X = S) Ru(II) complexes [21], while Jazzar et al. succeeded in obtaining two distinct Ru(II) isomers, owing to 2-substituted carboxy-pyrrolyl ligands acting as a model for catalytic intermediates in the Murai coupling reactions (B) [22]. Pyrrolide-bearing 2-substituents such as formyl, keto, carboxylate, sulfinyl, and sulfonyl have further been described in 2,2′-bipyridyl complexes by Lundrigan (C) [23]. Reactivity between the analogous RuHCl(CO)(PPh_3_)_3_ to achieve Ru-ester or imine complexes was also reported by Jui-Hsien Huang (D) [24]. In particular, isomers differing by mutual Ru-H/CO ligands have been selectively isolated by adopting distinct reaction conditions (E) [25]. All of them exhibit stable (N,O) or (N,N) chelation, while showing non-fluxional behavior, which can disclose interesting features to reveal effective biological activity. 

**Figure 2:**
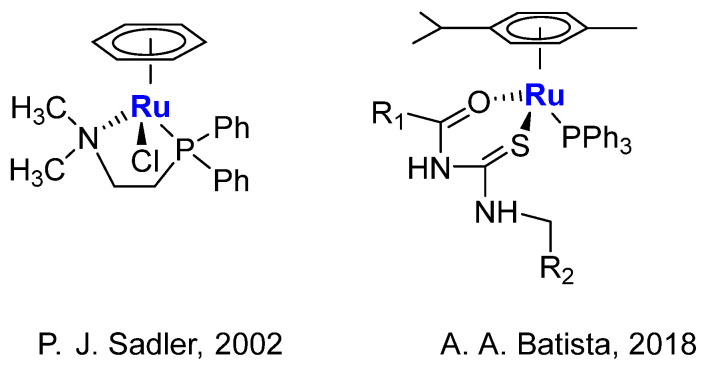
Example of phosphoamino-ligand that undergoes Ru-N cleavage, whereas upon hydrolysis, the thioacetamide chelate complex promotes exclusive S-coordination [27,30].

Multihapto coordination can be easily altered by changing the heteroatom chelation mode when complexes are exhibiting distorted geometries by steric constraints. Chelate opening has been described to occur in Cr, Cu, and Zn complexes induced by redox reactions directed to metal sites or bonded ligands [26] through hydrolysis [27], pH variation [28], or external ligand introduction [29]. The processes are frequently assisted by coordinative solvent inclusion, prior to ligand association. By way of example, Sadler reported rearrangement for Ru-species, in which the hemilabile phosphoamino-ligand undergoes Ru-N cleavage in solvent-induced reversible reactions [30], thus preferring soft P-coordination. Similarly, thioacetamide chelate Ru-complexes are reported to promote exclusive S-coordination upon hydrolysis treatment. Fluxional behavior results in opening chelate fragments, generating vacant coordination sites that are considered accountable for biological applications [27] (Figure 2).

In the current description, we report the coordination of pyrrole derivatives containing the carboxylic (**H_2_L_1_**) or aldehydic (**HL_2_**) group in [Ru(H)_2_CO(PPh_3_)_3_] **1**, by concomitantly substituting both the hydride and PPh_3_ ligands. The optimized reactions gave satisfactory yields of the expected products, affording both dihapto homoleptic k^2^(O,O)- and heteroleptic k^2^(N,O)- coordinative fashion. Carboxylate species kinetic mixtures have been spectroscopically evaluated under distinct reaction conditions and studied by computational methods. The intermolecular π-π stacking interactions and dipole H-binding intercept the related stabilized species **2**. An unusual change of coordination with a simultaneous solvent inclusion is obtained by refluxing **2** in acetonitrile. The impact of coordinated acetonitrile by biological assays was evaluated on the metallodrug’s efficacy as antibacterial. Two plausible mechanisms were proposed and supported by DFT calculations. Further, concerning the isolated Ruthenium species bearing pyrrole 2-aldehyde side arms, distinct geometric or functional isomers are shown by single crystal X-ray diffraction (SCXRD) data and supported through DFT computational analysis.

## 2. Results and Discussion

### 2.1. Syntheses of Complexes ***2***–***3***

**Scheme 1:**
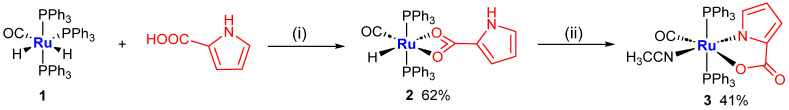
Synthesis of complexes **2** and **3**. (i): Refluxing 1,2-DME, 45 min, -H_2_, -PPh_3_; (ii): refluxing MeCN, 40 h, -H_2_. After purification, the overall yield of complex **3** with respect to the starting complex **1** is 41%.

The adopted procedure to form carboxy-derivative **2** involves the reaction of **1** with a stoichiometric amount of **H_2_L_1_** using a non-polar solvent such as 1,2 DME (Scheme 1), while k^2^(N,O)-species **3** was obtained in a satisfying yield by refluxing the chelate k^2^(O,O)- homoleptic derivative **2** in MeCN for 40 h.

### 2.2. Kinetic Mixture

**Figure 3:**
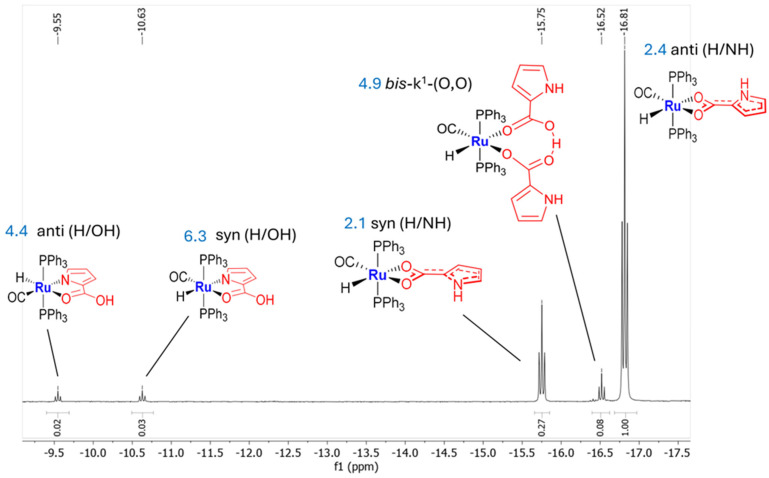
^1^H NMR hydride region in CDCl_3_ (from −9.0 to −17.5 ppm) of a kinetic mixture, obtained by running the reaction of **1** with **H_2_L_1_** through MW solicitation for 15 min in CPME, with the attributed geometries based on DFT energetic calculations. The latter were run using the def2-QZVPP basis and the M06 functional including CPME solvent effects and are reported in blue. The small upfield-shifted hydride resonances are assigned to heteroleptic k^2^(N,O)-derivatives, involving N-H pyrrolyl activation with chelation through the CO-carboxy function.

Interestingly, a kinetic mixture of products was formed by MW treatment within 15 min in cyclopentyl methyl ether (CPME) solvent. ^1^H and ^31^P NMR spectra (Figure 3, Supplementary Material Figure S7) suggest the formation of five distinct species, which are composed by two highfield-shifted homoleptic dihapto k^2^ (O,O)-carboxylate **2** and two k^2^(N,O)- heteroleptic isomers. In fact, DFT energetic calculations suggest a planar symmetric cycle as the ligand, supported by the small Ru-H triplet at δ = −16.52.

**Scheme 2:**
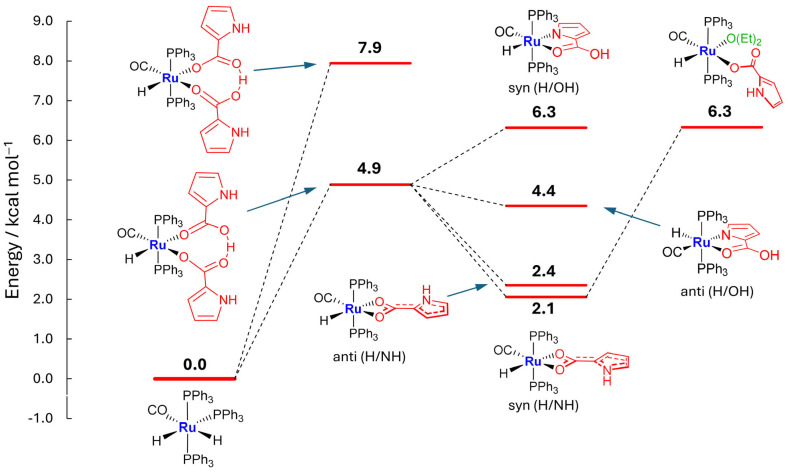
DFT calculations suggest a stable *syn*-k^2^(O,O)-**2** species found at lower energies and exhibiting on the same side pyrrole-NH and Ru-H, whose ^1^H NMR signals fall at 8.15 and −15.75 ppm, respectively. Finally, the thermodynamically stable isomers are assigned to N-pyrrolyl donor moiety *trans*-located to the electron-withdrawing CO ligand. The *anti* heteroleptic complex shows structures at relatively higher energies, and the corresponding signals disappear upon longer time running of kinetic mixtures. In spite of DFT-calculated low energy, the Et_2_O-coordinated species, which could be conceivably formed by elution through CH_2_Cl_2_/Et_2_O column chromatography or during numerous crystallization attempts, has never been spectroscopically intercepted. Arrows are used to show the energy of each structure.

Both k^2^(O,O)- and k^2^(N,O)- complexes could likely derive from the evolution of an elusive *bis*(k^1^)-pyrrole carboxy kinetic species (Scheme 2), as the dimeric nature of the coupled carboxylic acids is well known in solids and similarly is conceivable to occur in non-polar solvents [31,32]. So, concerning kinetic intermediates, the coupled pyrrolyl-carboxy acids could analogously form O-H…O hydrogen-bonded Ru-coordinated *bis*- k^1^-dimer.

By monitoring the kinetic mixture at room temperature for 60 h in CDCl_3_, the ^1^H NMR spectra show both *syn*- and *anti*- k^2^(O,O)- planar pyrrole isomers to exhibit greater stability, since their occurrence largely increases over time (Supplementary Material Figure S6). Two Ru-H small triplets at δ −9.55 and −10.63 (Figure 3), due to the equivalent phosphine coupling, are assigned to the (N,O)-chelate isomers [23], whereas adjacent downfield-shifted triplets at δ −15.75 (*syn*) and −16.81 (*anti*) are ascribed to k^2^(O,O)- homoleptic conformers, derived from a slightly restricted (carboxy)C-C(pyrrole) rotation barrier (11 Kcal mol^−1^).

### 2.3. Coordination of MeCN and Proposed Mechanisms **A** and **B**

**Figure 4:**
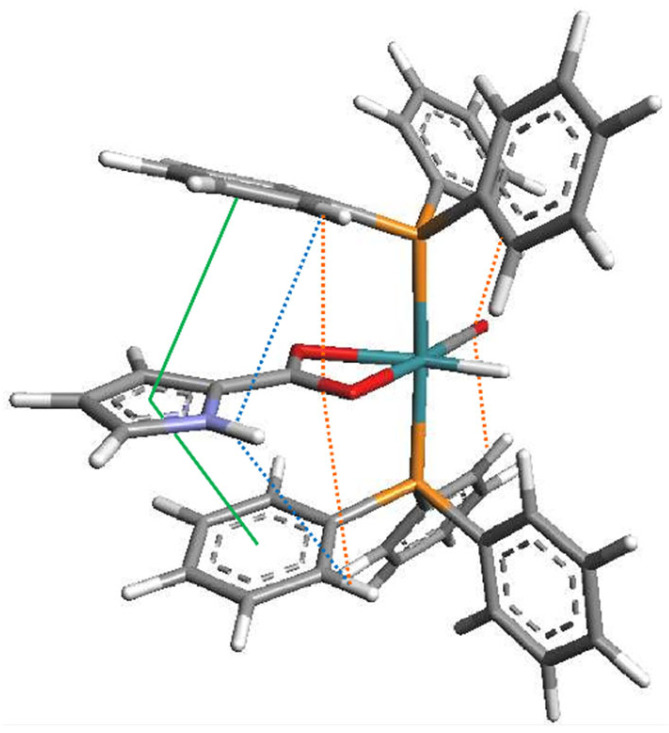
The calculated lowest energy configuration **2** (in vacuo, with def2-TZVP basis and the M06L functional) discloses NH-pyrrolyl and Ru-H units mutually on the same side, being further stabilized by intramolecular dipole interactions of Ph-CH/py-NH acid with basic pyCOO/Ru-CO moieties or notably π-π stacking interactions between pyrrole and phenyl planar rings. Dipole bindings were evaluated as effective if they were less than the sum of the Van der Waals radii [33] and are summarized as exemplary in Supplementary Material Table S1.

IR Ru-CO stretching at 1926 cm^−1^ with narrower carboxylate absorptions (1559, asym, 1461 sym, Δ = 98 cm^−1^) supports k^2^(O,O)-symmetrically coordinated **2**. On the other hand, at the IR Ru-CO peak of **3** at ν = 1944, largely separated COO-bands (ν = 1619 C=O, 1306 C-O D= 313 cm^−1^) together with NMR signals related to the ligated acetonitrile in CDCl_3_ [^1^H 1.35,^13^C 2.65 (Me), 125.82 (CN)] indicate transformation to the k^2^(N,O)-coordinative fashion mode (Scheme 1). The MeCN coordination is also relieved by the IR band at 1600 cm^−1^, which strongly supports the Me-C=N=Ru resonant structure, indicating strong backbonding participation. Exclusive occurrence of the ^1^H NMR signal at −15.75 ppm, assigned to the thermodynamic *syn*-(H/NH) dihapto k^2^(O,O)- **2** species (Figure 3), is likely stabilized by parallel π-π- stacking interactions between the pyrrolyl ring and PPh_3_ phenyl substituents or by the intramolecular short contacts between basic Ru-COO or -CO groups and the acidic Ph-CH or py-NH functionalities (see Figure 4).

**Scheme 3:**
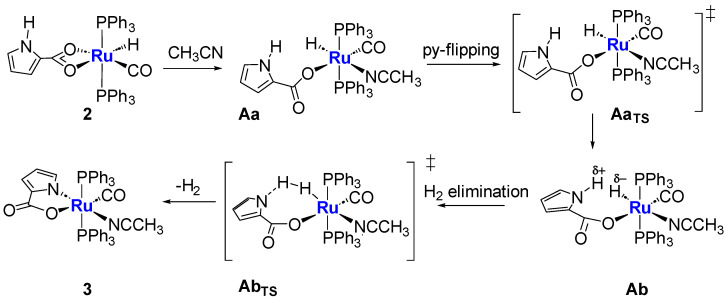
The promising mechanism **A** is proposed for explaining coordinative transformation from k^2^(O,O)-**2** to solvento-k^2^(N,O)- species **3**. The double dagger (‡) symbol is referred to transition states.

The plausible mechanism **A** is proposed (Scheme 3), involving facile k^2^(O,O)-ring opening by invoking a coordinating solvent approach, which prompts the cleavage of dihapto carboxylate, likely to be opposite to the strong *trans*-directing hydride ligand. Indeed DFT calculations support no evidence of the pentacoordinate commonly observed transient species, but point to a barrierless concomitant acetonitrile insertion. Subsequent twisting about the monohapto carboxy-pyrrole side arm results in the approach of pyrrolyl-NH to the Ru-H unit, thus promoting H_2_ release, upon sliding across PPh_3_ ligands. Pyrrolide ring closure ultimately provides heteroleptic k^2^(N,O)- solvento-species **3**.

**Scheme 4:**
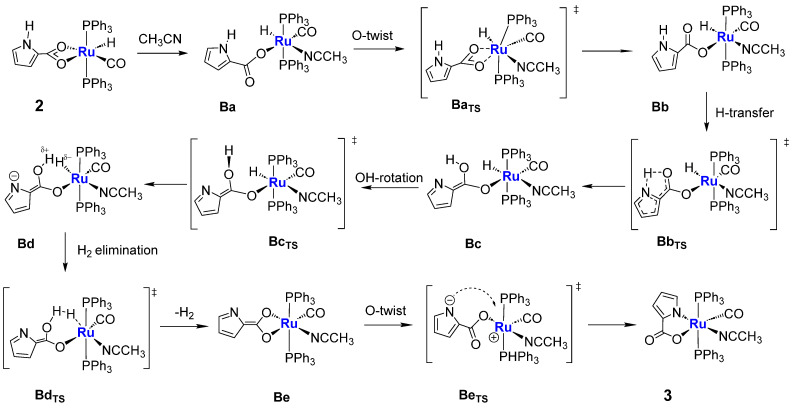
Proposed mechanism **B**. The double dagger (‡) symbol is referred to transition states.

An alternative reasonable mechanism **B** (Scheme 4), based on carboxyl assistance [34,35] to trigger H_2_ release, is further proposed, showing energy barriers slightly lower than the single transition state-determining mechanism **A**. As carboxy-pyrrole is twisting about the Ru-O bond, the exchange of oxygen atoms triggers proton transfer from NH-pyrrole to the carboxylic group, favoring “formation and releasing” of the dihydrogen, thus achieving pyrrolide ring closure.

**Figure 5:**
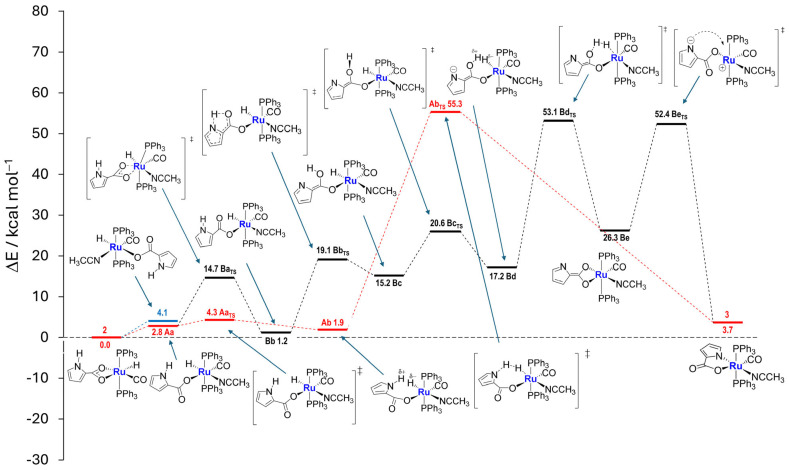
The proposed mechanisms are identified by differently colored paths (red for **A** and black for **B**). However, the blue-colored higher-energy step (4.1 kcal/mol) belongs to a never-observed isomer, showing the unfavorable opened configuration, with opposite H/C(O)O units. The double dagger (‡) symbol is referred to transition states.

The highest energy barriers (Figure 5), which involve carboxy-rotation and H-transfer, globally concerning H_2_ release, require prolonged refluxing acetonitrile (40 h) and constitute crucial paths for both the presented **A** and **B** mechanisms.

### 2.4. Syntheses of Complexes ***4***–***5***

**Scheme 5:**
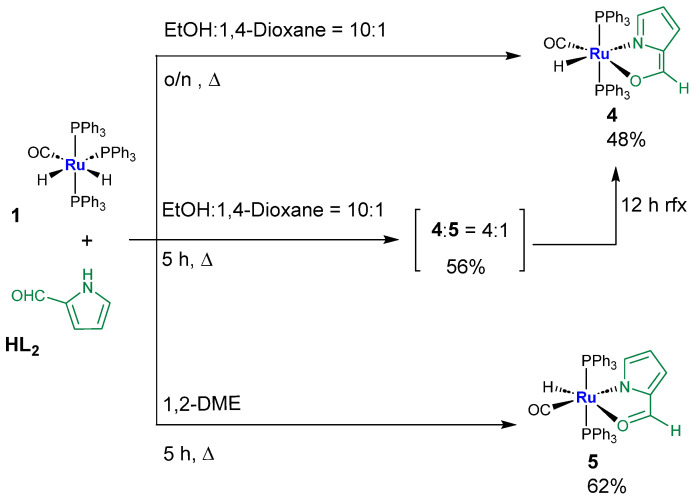
Different syntheses of complexes **4** and **5**.

By refluxing solvent mixtures with prevalent proportions of EtOH or with less polar 1,2-DME, the reaction of **1** and **HL_2_** selectively affords distinct **4** or **5** isomers, which are believed to be at equilibrium with the reciprocal stabilization greatly affected by the solvent polarity. Prolonged reflux (12 h) in a prevalently polar solvent mixture composed by EtOH: 1,4 dioxane = 10:1, selectively leads to complex **4** as the most stable (48% yield), whereas by running the reaction in non-polar 1,2-DME, the exclusive conversion to **5** is achieved (62%). By mixing solvents of different polarity (EtOH:1,4-Dioxane = 10:1) for 5 h, the reaction yields **4** + **5** isomers in a 4:1 proportion (Scheme 5). Both isomers exhibit pronounced antisymbiotic influence, which rules Ru-CO in *trans*-position to anionic groups, namely the O-ethenoyl unit in the case of **4**, or N-pyrrolide for the **5** species.

DFT calculations for **4** and **5** were performed in vacuo and in 1,2-DME and EtOH solvents. In vacuo, **5** results as the most stable by 1.7 kcal/mol. The energy difference reduces to 1.4 kcal/mol and to 0.6 kcal/mol by including 1,2-DME and EtOH solvent effects, respectively. We were not able to reproduce by DFT the exchange in stability observed experimentally, but the decrease of stability of **5** with respect to **4** in polar solvents is clearly evident, as shown in Supplementary Material Figure S37.

#### Aldehyde Isomer Description and Extended π−Aromaticity of Five-Membered Fused Rings

**Scheme 6:**
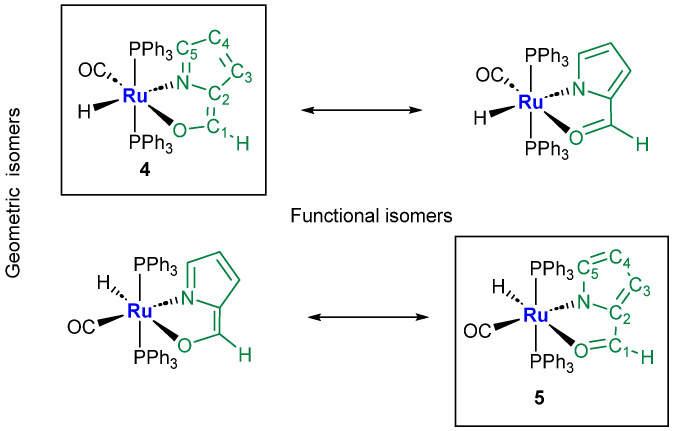
Proposed isomers of **4** and **5**. The most plausible isomer for each species is framed in the scheme.

The isolated ethenoyl-azafulvene **4** Ruthenium species together with the 2-formyl-pyrrolide isomer **5,** both framed in Scheme 6, show CO oppositely located to the corresponding anionic ligands. Charge delocalization suggests extended π-aromaticity along the fused planar five-membered rings for both isomers, thus supporting their stability.

**Figure 6:**
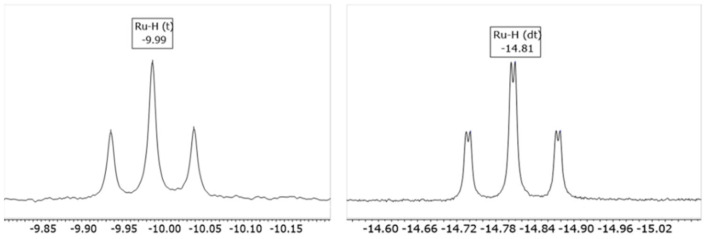
Comparison between the Ru-H ^1^H NMR signals of **4** and **5**. Besides the expected coupling with equivalent apical phosphines (^2^J_HP_ = 20.7 Hz), the multiplicity of **5** clearly evidences delocalization from *trans*-directing Ru-H to the coordinated carboxaldehyde ligand (^1^H NMR δ = 8.05 ppm, ^2^J_HP_ = 20.7, ^4^J_HH_ = 1.8 Hz).

The Ru-H NMR signal shows multiplet (t) at δ = −9.99 for **4**, due to the expected coupling with *trans*-phosphine ligands. However, in the case of **5** (Figure 6), the triplet centered at −14.81 ppm is further coupled with the CHO moiety (^4^J_HH_ = 1.8 Hz), as confirmed by COSY and selective HOMODEC NMR experiments (Supplementary Material Figures S32 and S33), which supports extended delocalization.

**Scheme 7:**
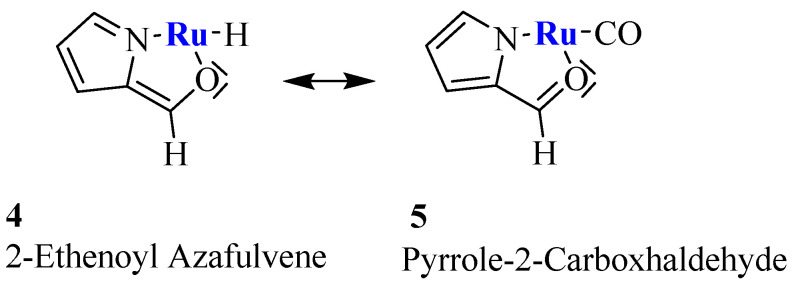
Resonant structures of **HL_2_**-Ru.

Notable spectroscopic considerations are in line with the proposed structures (Scheme 7):

- The IR Ru-H stretching appears at ν 1989 for **5**, while the related absorption results were overlapped by the strong Ru-CO at 1992 cm^−1^ in the case of **4**;
- As expected, the ^1^H NMR ethenoyl and the C^5^H imino-pyrrole signals of **4** fall at 7.57 and 6.84 ppm, respectively (Supplementary Material Figure S21), whereas the analogous carboxaldehyde and the pyrrolide C^5^H-unit resonances concerning complex **5** appear conversely shifted at δ 8.06 and 6.13 ppm (Supplementary Material Figure S29);
- The adjacent C5=N bond, measured by X-ray (1.29 Å) or evaluated by DFT calculations (MBO = 1.41), is slightly elongated in **4**; conversely, in the case of the Ru- carboxaldehyde pyrrole **5** species, the C5-N bond is calculated to be 1.33 Å with a bond order of 1.38 Å, showing a trend in accordance with the proposed isomers.

### 2.5. ESI-Ms Spectra

Complex **2** (Supplementary Material Figure S1) displays the expected signals corresponding to hydride or ligand (**HL_1_**) loss, at *m*/*z* 764 [M–H]^+^ and 655 [M-**HL_1_**]^+^, whereas peaks at 696 or 737 *m*/*z* are related to subsequent MeCN incorporation. Three peaks are revealed at 805 [M + H]^+^, 827 [M + Na]^+^ and at 843 [M + K]^+^ for the solvento complex **3** (Supplementary Material Figures S11 and S12), with significant fragmentations, namely greater than molecular peak, ascribed to **L_1_** ligand loss (696) and concomitant incorporation of a further acetonitrile molecule (737). Analogously, the same series of fragmented signals are observed for both **4** and **5** species (Supplementary Material Figures S19 and S27).

Since **2** gave crystals not suitable to be characterized by SCXRD, the HRMS spectrum was then acquired, showing the molecular signal at 764.1090, which assesses the proposed structure (Supplementary Material Figures S3 and S4).

### 2.6. Description of the X-ray Crystal Structure of ***3***–***5***

**Figure 7:**
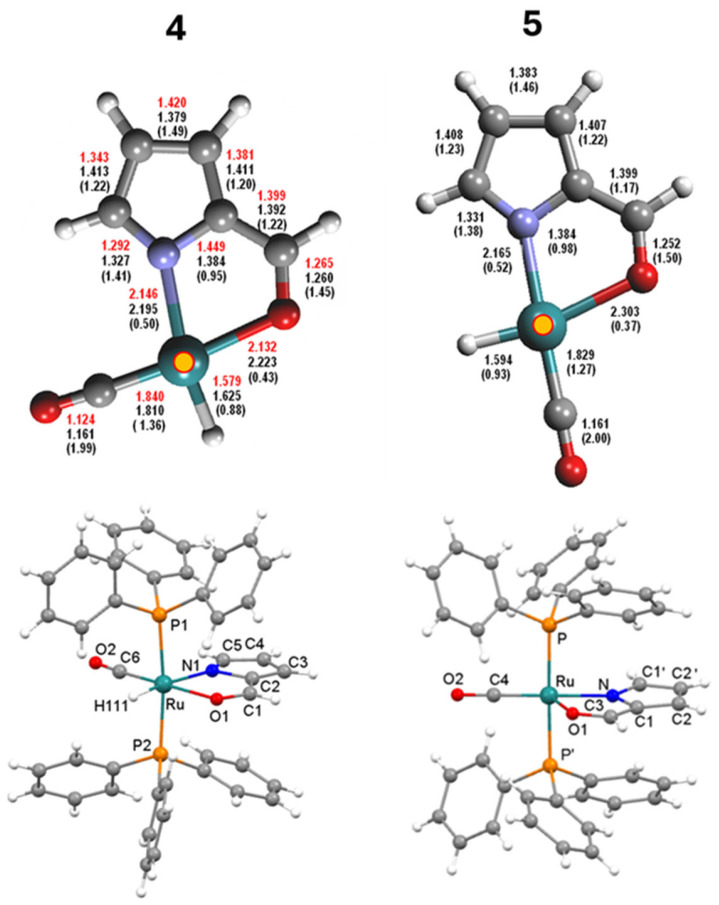
Single crystal structure of **4** and **5**. Experimental (red) and calculated (black) bond lengths are reported above for **4** and only calculated for **5**. Mayer Bond Orders are reported in brackets. In **5**, a twofold axis passing through O2, O4, Ru, N, and the C2-C2′ bond is present.

**Figure 8:**
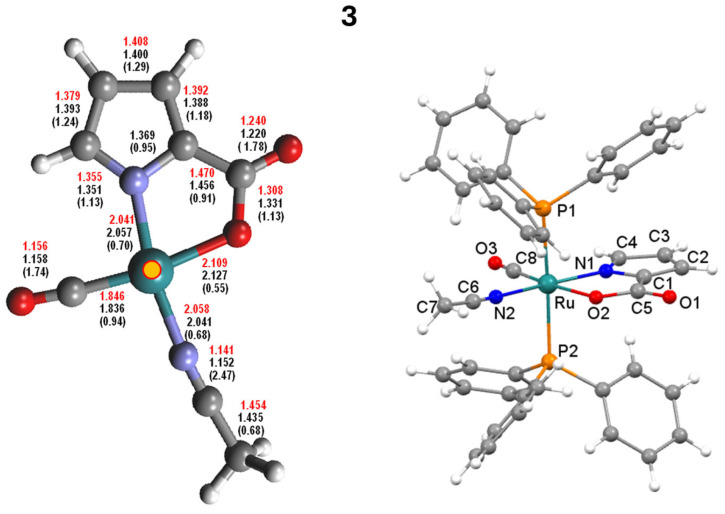
X-ray experimental (red) and DFT-calculated (black) bond lengths of **3**. Calculated Mayer Bond Orders are reported in brackets.

X-ray-quality crystals of **3**–**5** were grown by double-layer crystallization techniques (DCM–hexane = 1:10), and their structure was determined using SCXRD. In all three **3**–**5** complexes, the Ruthenium atom adopts a distorted octahedral geometry with the PPh_3_ ligands in a mutual *trans*-position (Figure 7 and Figure 8). In complex **3** (Figure 8), the coordination sphere of Ru is completed by one carbonyl, one MeCN, and one (N,O) chelate pyrrolide 2-carboxylate ligand. The N-atom of the MeCN ligand is *trans* to the N atom of the dianionic pyrrolide ligand, and the Ru-N distances are 2.058(4) and 2.041(4) Å for Ru-N2 (acetonitrile) and Ru-N1(pyr), respectively. The N1-Ru-O2 bite angle in the metallacycle ring [78.7(1)°] is in the range expected for this family of five-membered rings. The uncoordinated carboxylate oxygen O1 is engaged in intermolecular non-classical C-H…O hydrogen bonds (Supplementary Material Figure S39) with the methyl hydrogens of MeCN of adjacent molecules.

Although the crystallographically imposed twofold symmetry observed in the X-ray investigation of **5** (details to follow) precludes the possibility of inferring reliable considerations by a strict comparison of isomers **4** and **5**, we decided to report the comparison by experimental and DFT-calculated data for **4** and DFT-calculated data only for **5** (Figure 7, top).

In the isomers **4** and **5**, shown in Figure 7, the coordination sphere of the Ru atom is completed by one hydride, one CO, and a chelate (N,O) pyrrole carboxaldehyde, with the N-atom in *trans*-position to the hydride in **4** and to CO in **5** in addition to the PPh_3_ ligands in a mutual *trans*-position. In complex **5**, the molecule lies on a crystallographic twofold axis passing through O2, C4, Ru, and N and bisecting the C2-C2′ bond (symm op.: –x + 1, y, −z + 0.5) and as a consequence, the CHO group of the pyrrole is disordered exactly (50:50). The Ru-N distances are significantly different in the two complexes, being 2.146(5) Å in **4** and 2.07(1) Å in **5**, whereas the Ru-O bond lengths are inverted, being shorter in **4** [2.133(4) Å] with respect to the one in **5** [2.269(8) Å] as a consequence of the *trans*-effect exerted by the hydride ligand in the latter. Although the hydridic hydrogen was not located, its presence *trans*- to O1 in the equatorial plane of Ru can be inferred from the lengthening of the Ru-O1 interaction and other geometrical features. The N-Ru-O bite angles range from a typical value of 79.2(2)° for **4** to the narrow 67.8(2)° for **5**. These observations are in good agreement with the values reported for two similar *cis*- and *trans*- [Ru(PPh_3_)_2_(CO)(NC_4_H_3_C(O)CH_3_)H] isomers [22].

### 2.7. Antimicrobial Activity

Various pathogenic bacteria represent a frightening threat to humanity and modern healthcare. Ruthenium-based compounds exhibit antimicrobial activity against a wide range of Gram-negative and Gram-positive bacteria and fungi [36]. Numerous studies have explored the efficacy of pyrrole organic molecules as antimicrobial agents [18]. However, the investigation of Ruthenium (II) complexes containing pyrrole derivatives in this regard remains relatively limited and unexplored. Different concentrations of **2**, **3**, and **5** were used to determine their minimum inhibitory concentrations on a Gram-positive (*Staphylococcus aureus* ATCC 29213) and a Gram-negative (*Escherichia coli* ATCC 11105) bacteria and a fungal strain (Candida albicans SO1) by the turbidimetric technique in suspension. Compounds were tested in the range of 2–100 µg mL^−1^ (Supplementary Material Figures S45–S47). A comparison with the ligand **H_2_L_1_** and the Ru(II) precursor **1** was performed (Supplementary Material Figures S49 and S50). Complex **4** was not tested because of its low solubility in water. The growth of *E. coli* and *C. albicans* was not affected by any of the compounds tested. A different response was obtained when the same test was performed on the Gram-positive *S. aureus*. In particular, while **2** and **5** did not affect bacterial growth, **3** reduced *S. aureus* growth and exhibited a minimum inhibitory concentration of 50 µg mL^−1^ (Supplementary Material Figure S48). It is noteworthy that both species **2** and **3** are Ru(II) complexes with pyrrole-2-carboxylic acid are chelated to the metal center. The enhanced activity of **3** could be ascribed from the k^2^(O,O)- to k^2^(N,O)- shift of the coordination mode and to the susceptibility of the solvate complex.

## 3. Materials and Methods

### 3.1. General

All reactions were routinely carried out under argon atmosphere, using standard Schlenk techniques. Solvents were HPLC grade and degassed before use. Glassware was oven dried before use. Infrared spectra (4000–400 cm^−1^) were recorded at 298 K on a PerkinElmer Spectrum 2000 FT-IR (Fourier transform infrared) spectrophotometer (Waltham, MA, USA), and ESI MS (electrospray ionization mass spectrometry) spectra were recorded on a Waters Micromass ZQ 4000 (Milford, MA, USA), with samples dissolved in CH_3_OH or MeCN. The spectra acquired for the powders were compared to those obtained for the crystals, and no significant differences were observed.

The HRMS spectrum of **2** was recorded with an (Waters USA) Esi TOF. All deuterated solvents were degassed before use. NMR measurements were taken at 298 on Varian Inova 300 (Varian, Palo Alto, CA, USA), Mercury Plus 400 (Oxford Instruments, Abingdon-on-Thames, UK), and Inova 600 (Varian, Palo Alto, CA, USA) instruments. Frequencies are reported in Hz, and the chemical shifts are referenced to the solvent. The chemical shifts are expressed in parts per million (ppm). The multiplet lists are reported with the aid of two-dimensional COSY, HSQC, and HMBC NMR spectra. All the chemicals were of reagent grade and were used as received from commercial suppliers. Commercially available [RuCl_3_·xH_2_O] was purchased from Strem (Bischheim, France). Pyrrole derivatives were purchased from Aldrich (Darmstad, Germany). Compound [Ru(H)_2_(CO)(PPh_3_)_3_] was prepared following published methods [37].

### 3.2. Synthesis of k^2^(O,O)-[RuH(CO)(HL_1_)(PPh_3_)_2_], ***2***

Pyrrole-2-carboxylic acid **H_1_L_2_** (48 mg, 0.436 mmol) and [Ru(H)_2_(CO)(PPh_3_)_3_] (401 mg, 0.436 mmol) were dissolved in 1,2-dimetoxy ethane (25 mL) and refluxed until the IR Ru-CO absorption of **1** at 1940 cm^−1^ disappeared. After 45 min, the solvent was reduced to a minimum volume by vacuum pump, and 20 mL of n-hexane was added to precipitate a brown solid. The solid was filtered, washed with n-hexane (3 times with 10 mL aliquots) and methanol (3 times with 2 mL aliquots), and dried under vacuum.

Yield 62%. ATR-FTIR (ν, cm^−1^): 3302 (NH), 3056 (aromatic CH stretch), 2022 (RuH), 1926 (C≡O), 1559 (asym. COO), 1461 (sym. COO), 1434 (CH, PPh3), 1095 (PPh), 692 (Ph).^1^H NMR (400 MHz, CDCl_3_) (δ, ppm): 8.15 (1H, NH, s), 7.55–7.31 (30H, PPh3, m), 6.39 (1H, CH, bs), 6.11 (1H, CH, bs), 5.83 (1H, CH, bs), −16.74 (1H, t, Ru-H, ^2^J_HP_ = 20.1 Hz). ^13^C NMR (101 MHz, CD_2_Cl_2_) (δ, ppm): 204.92 (t, Ru-CO, ^2^J_CP_ = 14.2 Hz), 174.27 (COO), 134.49–128.21 (PPh_3_), 120.06 (C-H), 112.03 (C-H), 110.97 (C-H), 108.59 (C-H). ^31^P NMR (162 MHz, CDCl_3_) (δ, ppm): 44.38 (d, PPh_3_, ^2^J_PH_ = 18.5 Hz). ESI-MS^+^ (MeCN): (*m*/*z*): 764 [M–H]^+^.

Kinetic mixture. The reaction of **1** (196 mg, 0.213 mmol) with 1.3 eq of **H_2_L_1_** (excess 31 mg, 0.279 mol) was run under MW for 15 min in refluxing CPME. The colored mixture, which turned to dark brown solution, was dried under vacuum; then, the solid was filtered on celite and washed with Et_2_O (5 times with 20 mL aliquots) until the solution become colorless. The solid was then dissolved and extracted from the celite by eluting three times with a solvent mixture of CH_2_Cl_2_: Et2O = 2:1. The solvent mixture was evaporated under vacuum to give a dark powder. Yield of total product was 37.7% based on *w*/*w*. The reaction was repeatable to obtain a comparable ratio of products. As the reaction was run for longer times, the number of species decreased until only one was obtained.

### 3.3. Synthesis of k^2^(N,O)-[Ru(MeCN)(CO)(L_1_)(PPh_3_)_2_], ***3***

The complex [RuH(CO)(PyrCOO)(PPh_3_)_2_] **2** (95 mg, 0.124 mmol) was dissolved in acetonitrile (30 mL), and the solution was refluxed for 40 h. The solvent was then evaporated under reduced pressure, and the residue was dissolved in 2 mL of CH_2_Cl_2_ and precipitated with 10 mL of diethyl ether. The brown solid was filtered, washed with diethyl ether (3 times with 10 mL aliquots), and dried. Crystallization by DCM layered with hexane gave crystals suitable for X-ray diffraction studies.

Yield 67%. ATR-FTIR (ν, cm^−1^): 3047 (arom CH stretch), 2960 (Me CH stretch), 1944 (C≡O), 1619 (asym. COO), 1600 (CH Ph) 1481 (sym. COO), 1434 (CH stretch), 1306 (CNC py) 1095 (PPh), 695 (PPh_3_). ^1^H NMR (400 Mhz, CDCl_3_) (δ, ppm): 7.40–7.14 (30H, PPh_3_, m), 6.22 (1H, d, C-H, J = 3.8 Hz), 6.14 (1H, CH, bs), 5.90 (1H, C-H, d, J = 5.2 Hz), 1.35 (3 H, Ru-MeCN, s). ^13^C NMR (101 MHz, CDCl_3_) (δ, ppm): 202.89 (t, ^2^J_CP_ = 10.4 Hz, CO), 172.93 (Ru-COO), 132.36 (C-H), 137.43–123.27 (m, PPh_3_), 134. 16 (C-H), 132.36 (C-H), 125.82 (-CN), 113.82 (C-H), 111.25 (C-H), 2.65 (CH_3_). ^31^P NMR (162 MHz, CDCl_3_) (δ, ppm): 26.53 (s, PPh_3_, 2P). ESI-MS^+^ (MeOH): (*m*/*z*): 805 [M + H]^+^, 827 [M + Na]^+^, 843 [M + K]^+^.

### 3.4. Synthesis of k^2^(N,O)-[RuH(CO)(HL_2_)(PPh_3_)_2_], ***4***

Pyrrole-2-carboxaldehyde (26 mg, 0.27 mmol) and [Ru(H)_2_(CO)(PPh_3_)_3_] (250 mg, 0.27 mmol) were dissolved in a 10:1 mixture of Ethanol (20 mL) and 1,4-Dioxane (2 mL) and refluxed overnight. After cooling to room temperature, the light-brown solid was filtered. The powder was recrystallized in diethyl ether and pentane mixture, filtered, and washed with methanol (2 times with 2 mL aliquots) and pentane (3 times with 10 mL aliquots). Crystallization by DCM layered with hexane gave crystals suitable for X-ray diffraction studies.

Yield 48%. ATR-FTIR (ν, cm−1): 3054 (aromatic CH stretch), 1922 (C≡O), 1563 (C=O), 1481 (CH pyr) 1431 (CH Ph), 1299 (CNC), 1091 (PPh), 741 (PPh_3_). ^1^H NMR (400 MHz, CDCl_3_) (δ, ppm): 7.57 (s, H-C=O, 1H), 7.49 –7.22 (m, PPh_3_, 30H), 6.84 (s, C-H, 1H), 6.55 (d, C-H, J = 4.0 Hz, 1H), 6.04 (d, C-H, J = 3.9 Hz, 1H), −9.99 (t, Ru-H, ^2^J_HP_ = 20.5 Hz, 1H). ^13^C NMR (101 MHz, CDCl_3_) (δ, ppm): 206.66 (t, Ru-CO ^2^J_CP_ = 14.6 Hz), 177.40 (H-C=O), 144.76 (C-H), 143.08 (C-H), 134.16 (PPh_3_), 129.37 (PPh_3_), 127.83 (PPh_3_), 121.98 (C-H), 116.42 (C-H). ^31^P NMR (162 MHz, CDCl_3_) (δ, ppm): 46.16 (s, PPh_3_, 2P). ESI-MS+ (MeCN): (*m*/*z*): 748 [M–H]^+^.

### 3.5. Synthesis of k^2^(N,O)-[RuH(CO)(HL_2_)(PPh_3_)_2_], ***5***

Pyrrole-2-carboxaldehyde, **HL_2_** (52 mg, 0.54 mmol) and [Ru(H)_2_(CO)(PPh_3_)_3_] (500 mg, 0.54 mmol) were dissolved in 1,2-DME (20 mL) and refluxed until Ru-CO IR absorption of **1** at 1941 cm^−1^ disappeared. After 5 h, the solvent was evaporated under reduced pressure, and the residue was dissolved in 1 mL of CH_2_Cl_2_, giving light-brown powdered precipitates after addition of cold n-pentane aliquot (20 mL). The solid was then filtered, washed with n-pentane (3 times with 10 mL aliquots), methanol (2 times with 2 mL aliquots), and with n-pentane again (3 times with 10 mL aliquots) and finally dried.

Yield: 62%. ATR-FTIR (ν, cm^−1^): 3054 (aromatic CH stretch), 1998 (RuH), 1921 (C≡O), 1559 (C=O), 1480 (CH pyr) 1432 (CH Ph), 1311 (CNC), 1092 (PPh_3_), 743 (PPh_3_). ^1^H NMR (400 Mhz, CDCl_3_) (δ, ppm): 8.06 (s, H-C=O, 1H), 7.38–7.18 (m, PPh_3_, 30H), 6.51 (dd, C-H, J = 3.8, 1.0 Hz, 1H), 6.13 (d, C-H, J = 1.1 Hz, 1H), 5.80 (dd, C-H, J = 4.0, 1.4 Hz, 1H), −14.81 (td, Ru-H, J = 20.7, 1.8 Hz, 1H). ^13^C NMR (101 MHz, CDCl_3_) (δ, ppm): 204.20 (t, Ru-CO ^2^J_CP_ = 14.3 Hz), 178.18 (H-C=O), 143.85 (C-H), 143.51 (C-H), 134.13 (PPh_3_), 133.67 (PPh_3_), 129.38 (PPh_3_), 127.91 (PPh_3_), 121.70 (C-H), 115.96 (C-H). ^31^P NMR (121 MHz, CDCl_3_) (δ, ppm): 46.34 (s, PPh_3_, 2P). ESI-MS+ (MeCN): (*m*/*z*): 748 [M–H]^+^.

### 3.6. Synthesis of the Mixture of Isomers ***4*** and ***5***

Pyrrole-2-carboxaldehyde **H_1_L_2_** (26 mg, 0.27 mmol) and [Ru(H)_2_(CO)(PPh_3_)_3_] (250 mg, 0.27 mmol) were dissolved in a 10:1 mixture of Ethanol (20 mL) and 1,4-Dioxane (2 mL) and refluxed for 5 h. The obtained light-brown fine-powdered solid was filtered. The powder was then crystallized from diethyl ether and pentane mixture, filtered, and washed with methanol (2 times with 2 mL aliquots) and pentane (3 times with 10 mL aliquots). Yield: 56%.

### 3.7. Computational Details

DFT calculations were performed using the ORCA 4.2.1 suite of quantum chemistry programs [38]. Geometries were optimized using the M06L functional [39] and the def2-TZVP basis [40]. Dispersion corrections were accounted according to the DFT-D3 procedure (with zero damping functions), as suggested by Grimme et al. [41]. Vibrational frequencies were calculated at the optimized geometries, to check the stability of the stationary points and to evaluate the vibrational contribution to free energy. Free energies at the normal boiling temperature of the various solvents were evaluated by applying a scale factor of 0.9824 to the vibrational frequencies, adequate for the present combination of the DFT functional and basis set [42]. Fictitious pressures of 558 atm (acetonitrile), 494 atm (ethanol), 283 atm (dimethoxyethane), and 268 atm (CPME) were set for the various solvents, to account for the overestimation of entropic contributions [43]. Accurate single point energy calculations were performed at the def2-TZVP/M06L optimized geometries, with the large def2-QZVPP basis [40] and the M06 functional [44], with the inclusion of solvation effects of the proper solvent through the SMD model [45] and of dispersion interactions [41]. The final free energy of each structure, used to evaluate the relative free energies of the various products, was built by summing the difference between the def2-TZVP electronic and free energies with the def2-QZVPP single point electronic energy.

### 3.8. Crystallography

The X-ray intensity data for **3** and **5** were collected on a Bruker APEX-III CCD diffractometer using Cu−Kα radiation, whereas complex **4** was collected on an Xcalibur, Sapphire3 CCD diffractometer using Mo-Kα radiation. All data were processed using the Bruker suite of programs [46,47,48] for **3**, **5** or with the CrysAlis PRO software [49] for **4**, and all structures were solved by direct methods and refined with the SHELX program suite [50,51]. All non-hydrogen atoms were assigned anisotropic displacement parameters. Most of the hydrogen atoms, except the hydride in **5**, were located in the Fourier map, placed in idealized positions, and included as riding with constrained isotropic displacement parameters (C– H = 0.98, 0.95 Å for methyl and aromatic protons, respectively, and refined as riding with Uiso(H) = 1.5 or 1.2Ueq(C)). Molecular graphics were generated using the Mercury program [52]. Supplementary Material Table S2 reports crystal data and refinement parameters for **3**–**5**.

### 3.9. Antimicrobial Assay

Antimicrobial activity of Ruthenium compounds was tested against representative bacterial (*Staphylococcus aureus* ATCC 29213, *Escherichia coli* ATCC 11105) and fungal (*Candida albicans* SO1) strains. *S. aureus* and *E. coli* were grown in Nutrient broth, and C. albicans was grown in Sabouraud Dextrose broth (Becton, Dickinson and Co., NJ, USA). Microbes were inoculated in 96-well plates at 106 CFU/mL with or without different concentrations of Ruthenium compounds (2–100 μg mL^−1^) to evaluate minimum inhibitory concentrations. Microbial growth was followed by turbidimetric analysis, registering the Abs at 600 nm, after 24 h of incubation at 37 °C (EnSpire Plate Reader, PerkinElmer, MA, USA). Minimum inhibitory concentrations correspond to the lowest concentration of drug that abolishes microbial growth.

## 4. Conclusions

Four different Ruthenium (II) complexes, having a pyrrole ring functionalized by carboxylate (**HL_1_**) k^2^(O,O)-[RuH(CO)HL_1_(PPh_3_)_2_], k^2^(N,O)-[Ru(MeCN)(CO)(L_1_)(PPh_3_)_2_], or carboxaldehyde (**L_2_**) functions k^2^(N,O)-[RuH(CO)L_2_(PPh3)_2_], were synthesized and fully characterized, showing respectively a homoleptic (O,O)- or heteroleptic (N,O)- bidentate fashion mode. Up to three distinct k^2^(O,O)-conformers were observed in the kinetic mixture of species **2**, according to the DFT-calculated energy of restricted rotation around the Ru-carboxylate bond (11.4 kcal/mol), and intercepted by the intramolecular interactions, ascribable mainly to π-π stacking between heterocycle and phosphine phenyl rings, but also to the London interactions of pyrrole-NH or CH-phenyl acidic substituents with the COO-carboxylate or Ru-CO groups. Upon prolonged acetonitrile refluxing, the coordinated 2-carboxyl-pyrrole moiety exhibited unprecedented transformation from homoleptic k^2^(O,O)- to k^2^(N,O)- dihapto coordination, leaving the Ru-oxidation state through inclusion of the MeCN molecule. Selective syntheses to k^2^(N,O)-[RuH(CO)L_2_(PPh_3_)_2_] **4**, **5** isomers was accomplished by refluxing **1** with pyrrole aldehyde **HL_2_** in solvents of distinct polarity. According to Pearson’s anti-symbiotic selection, hard N- or O-hetero-anionic functions both show a *trans*-CO ligand, thus favoring back-donation and stability. In the case of the stable azafulvene **4**, the *trans*-directing Ru-hydride unit likely affects elongation of the opposite N-pyrrole (2.146 Å). X-ray crystallographic studies confirmed the structures of the **3**–**5** species. In this context, theoretical DFT investigations on the proposed mechanisms were run to exploit the unexpected coordinative modifications. As an application for this unprecedented behavior, a preliminary antimicrobial test using Ruthenium compounds revealed activity against *S. aureus*, exclusively in the case of the Ru-acetonitrile species **3**, whose antibacterial activity may be reasonably related to coordination vacancy. Further explorations on synthesizing analogously Ru-coordinated functionalized indole species are currently under investigation to examine possible anticancer applications as future perspectives.

## Data Availability

The original contributions presented in the study are included in the article/supplementary material, further inquiries can be directed to the corresponding author/s.

## Supplementary Materials

The following supporting information can be downloaded at: https://www.mdpi.com/article/10.3390/molecules29133068/s1, Characterization of complexes: Figures S1–S34; DFT calculation data: Figures S35 and S36, Table S1; X-ray Diffraction studies: Table S2, Figures S37–S43; Antimicrobial evaluation: Figures S44–S50.
