# Supplementary material for: Unpredictable Dynamic Behaviour of Ruthenium Chelate Pyrrole Derivatives
Source: Molecules. 2024 Jun 27;29(13):3068. doi: 10.3390/molecules29133068 (PMC11242957; doi:10.3390/molecules29133068)
Supplement: Supplementary file 1 [file molecules-29-03068-s001.zip › molecules-3072570-supplementary.pdf]

Supplementary Material

Unpredictable Dynamic Behavior of Ruthenium Chelate Pyrrole Derivatives

Giacomo Drius [1], Riccardo Tarroni [1,*], Matteo Birchmeier [1], Carola Parolin [2], Carla Boga [1], Magda Monari [3] and Silvia Bordoni [1,4,*]

[1] Department of Industrial Chemistry 'Toso Montanari', Alma Mater Studiorum, Università di Bologna, Via Piero Gobetti, 85, 40129Bologna, Italy; giacomo.drius2@unibo.it (G.D.); matteo.birchmeier@studio.unibo.it (M.B.); carla.boga@unibo.it (C.B.)

[2] Department of Pharmacy and Biotechnology, University of Bologna, 40127 Bologna, Italy; carola.parolin@unibo.it

[3] Department of Chemistry 'Giacomo Ciamician', Alma Mater Studiorum, Università di Bologna, Via Selmi 2, 40126 Bologna, Italy; magda.monari@unibo.it

[4] Health Sciences and Technologies Interdepartmental Centre for Industrial Research (CIRI SDV), University of Bologna, 40126 Bologna, Italy

* Correspondence: riccardo.tarroni@unibo.it (R.T.); silvia.bordoni@unibo.it (S.B.)

Table of Contents

Abbreviations 2

CHARACTERIZATION OF THE COMPLEXES 3

Characterization of **2** 3

  Mass spectra of **2** 3

  IR spectrum of **2** 5

  NMR spectra of **2** 6

Characterization of **3** 9

  Mass spectra of **3** 9

  IR spectrum of **3** 10

  NMR spectra of **3** 11

Characterization of **4** 15

  Mass spectrum of **4** 15

  IR spectrum of **4** 16

  NMR spectra of **4** 17

Characterization of **5** 21

  Mass Spectrum of **5** 21

  IR spectrum of **5** 22

  NMR spectra of **5** 22

DFT CALCULATIONS 28

Mayer Bond Orders 28

  MBO complex **2** 28

X-RAY DIFFRACTION STUDIES 29

  Crystal Data 29

Crystal Packing of **3** 30
Crystal Packing of **4** 30
Antimicrobial evaluation 33
Antimicrobial tests on Candida albicans 33
Antimicrobial tests on Escherichia coli 34
Antimicrobial tests on Staphylococcus aureus 35

Abbreviations

SCXRD	Single Crystal X-ray Diffraction
DFT	Density Functional Theory
PyrCOO	Pyrrole-2-carboxylic acid
PyrCHO	Pyrrole-2-carboxaldehyde
CPME	Cyclopentyl Methyl Ether
MeCN	Acetonitrile
1,2-DME	1,2-Dimetoxyethane
MW	Microwave
$CDCl_3$	Deuterated Chloroform
CD_2Cl_2	Deuterated Dicloromethane
EtOH	Ethanol
DMSO	Dimethyl sulfoxide
Et_2O	Diethyl Ether

CHARACTERIZATION OF THE COMPLEXES

Characterization of **2**

Mass spectra of **2**

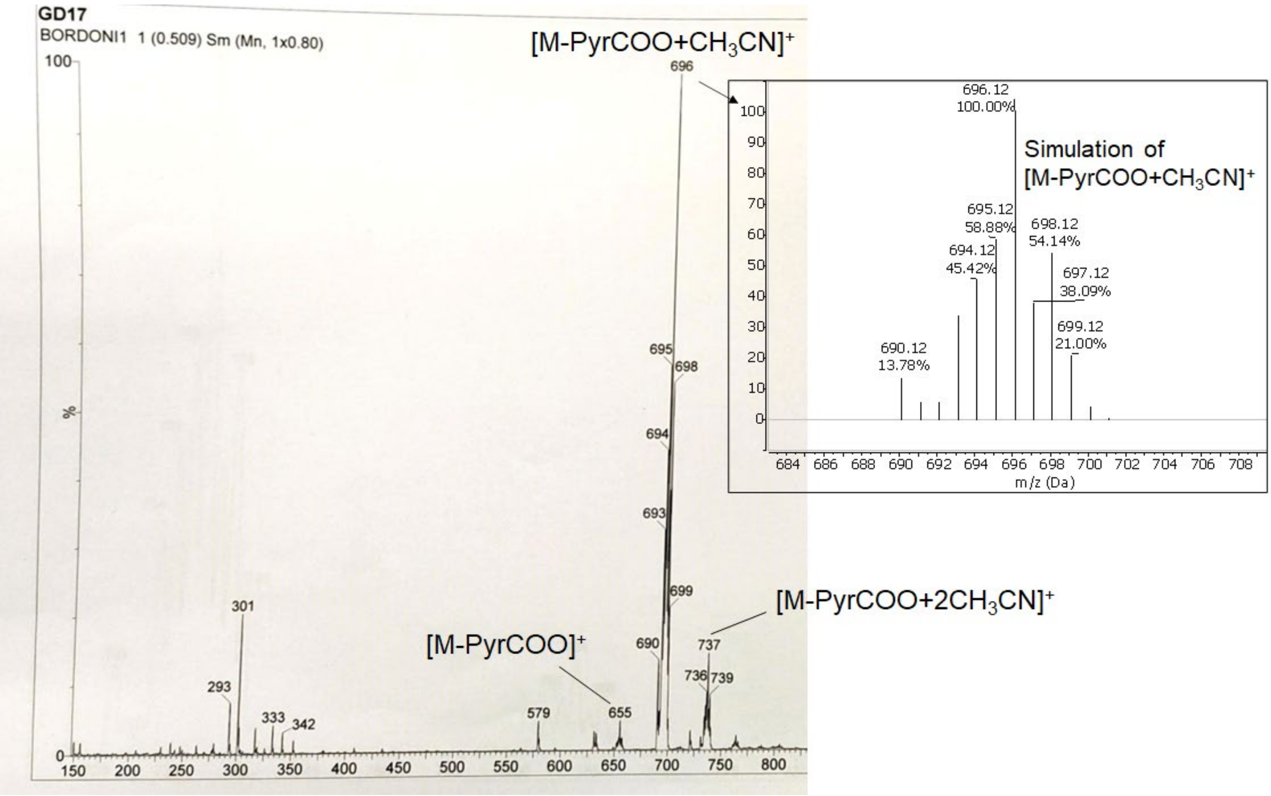

Figure S1: ESI-Mass spectrum of **2** (positive mode, m/z: 150 – 850) in CH_3CN.

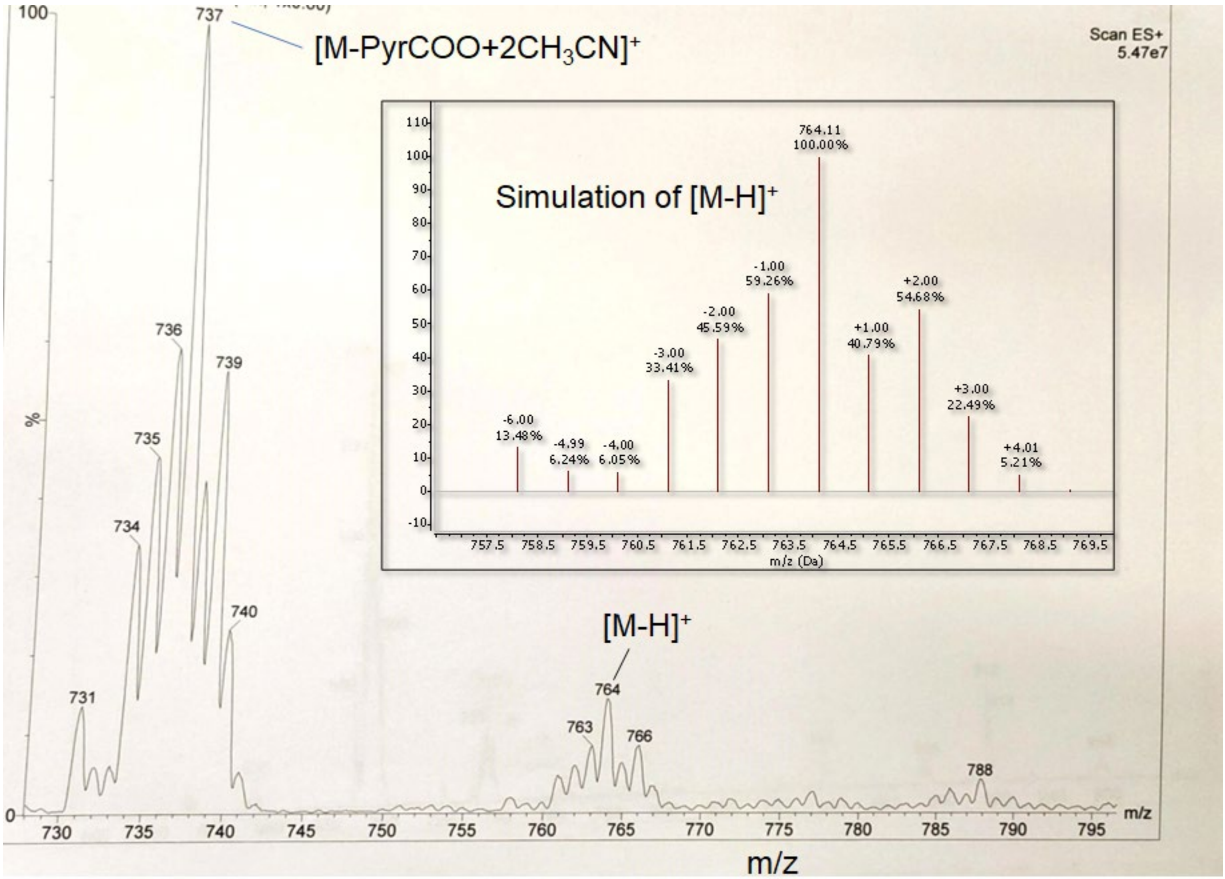

Figure S2: ESI-Mass spectrum of **2** (positive mode, m/z: 730 – 795) in CH_3CN.

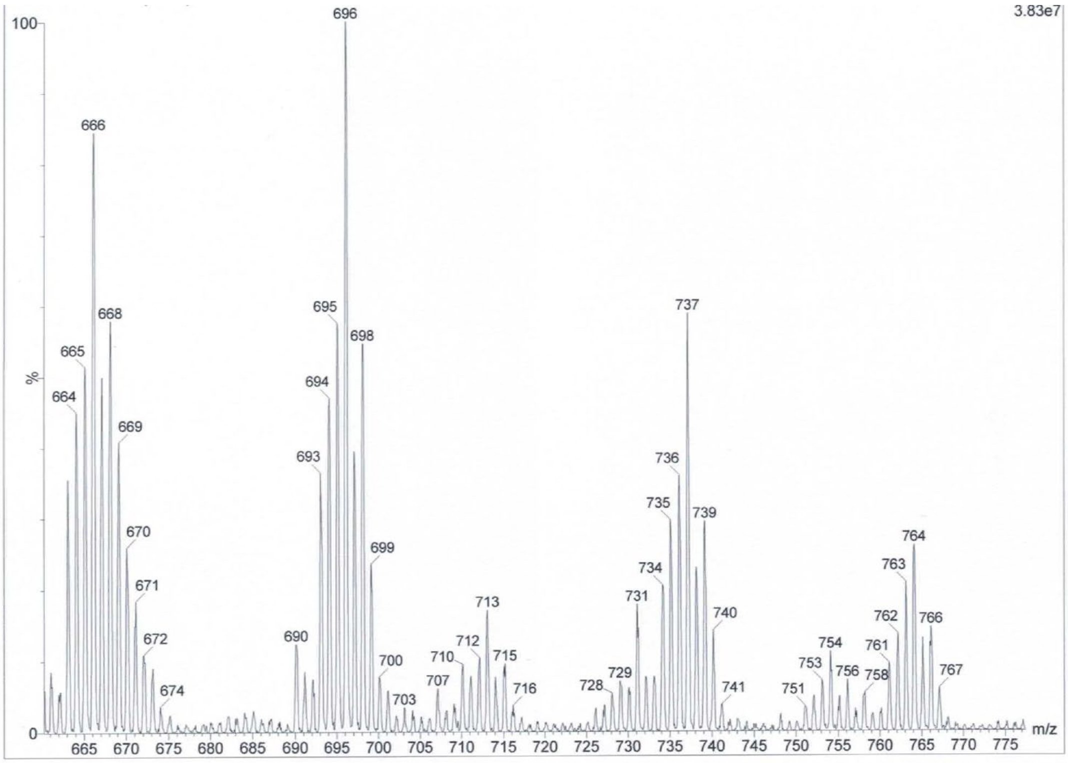

Figure S3: ESI-HRMS spectrum of **2**. (positive mode, m/z: 665 – 775) in CH_3CN.

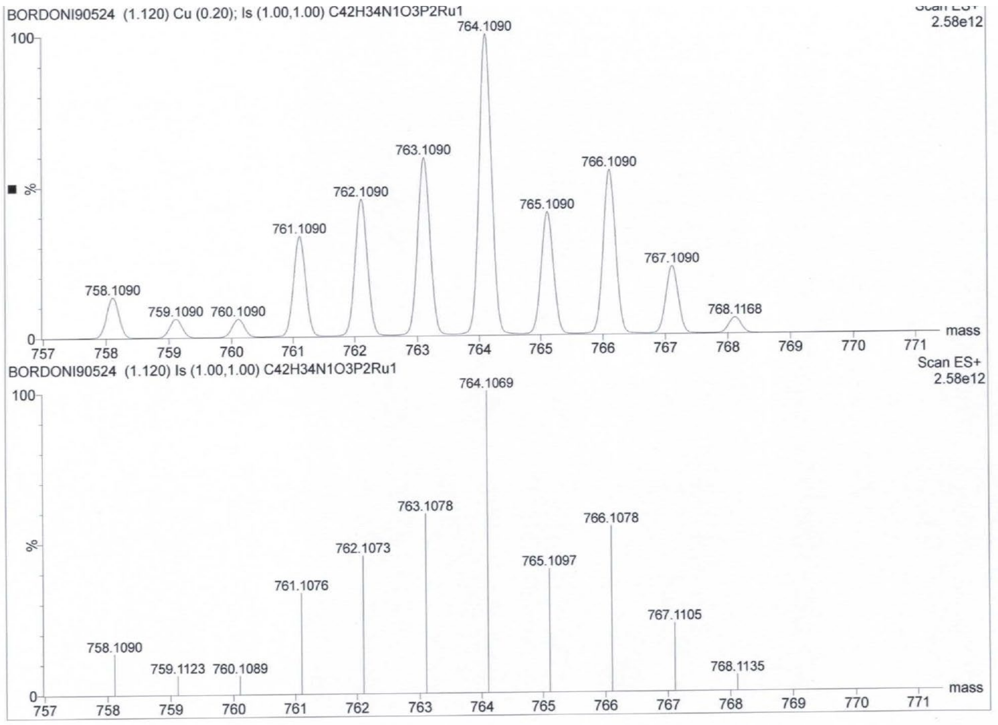

Figure S4: ESI-Mass spectrum of **2** (positive mode, m/z: 757– 771) in CH_3CN compared to simulation.

IR spectrum of 2

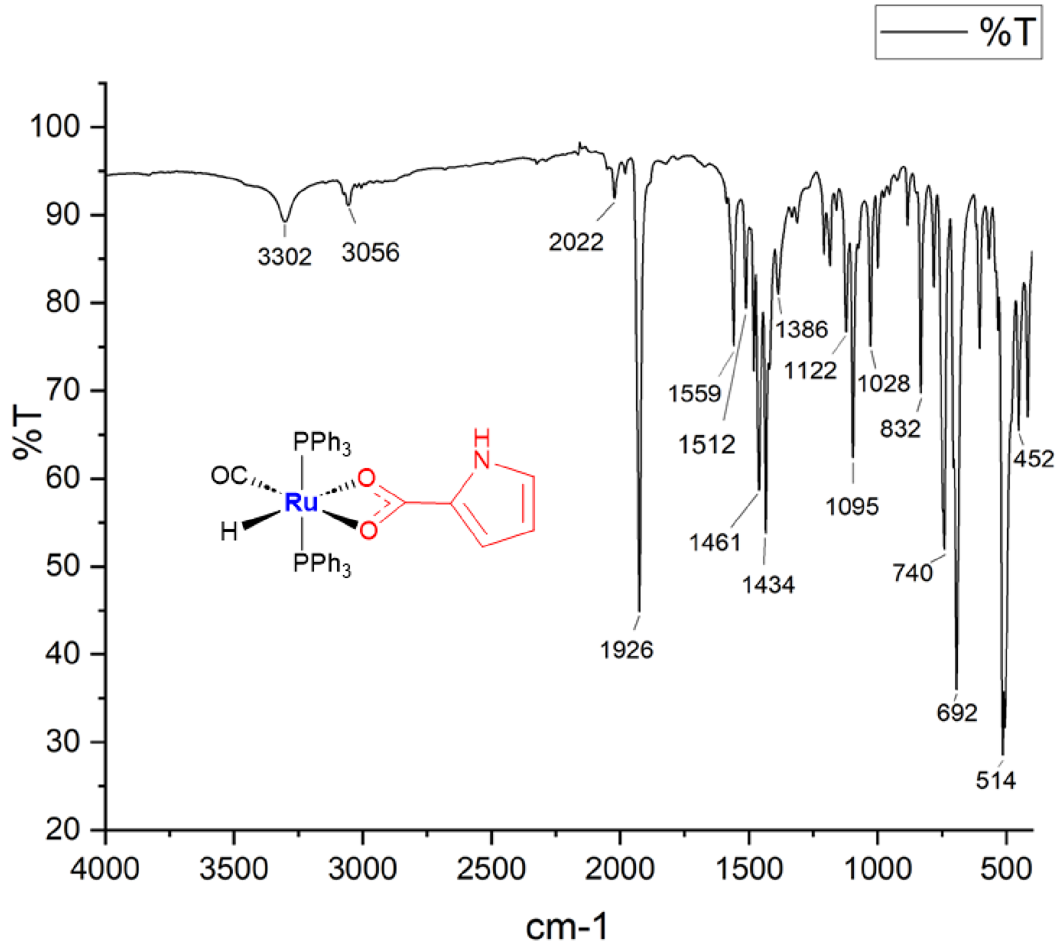

Figure S5: IR spectrum of **2** (ATR).

NMR spectra of **2**

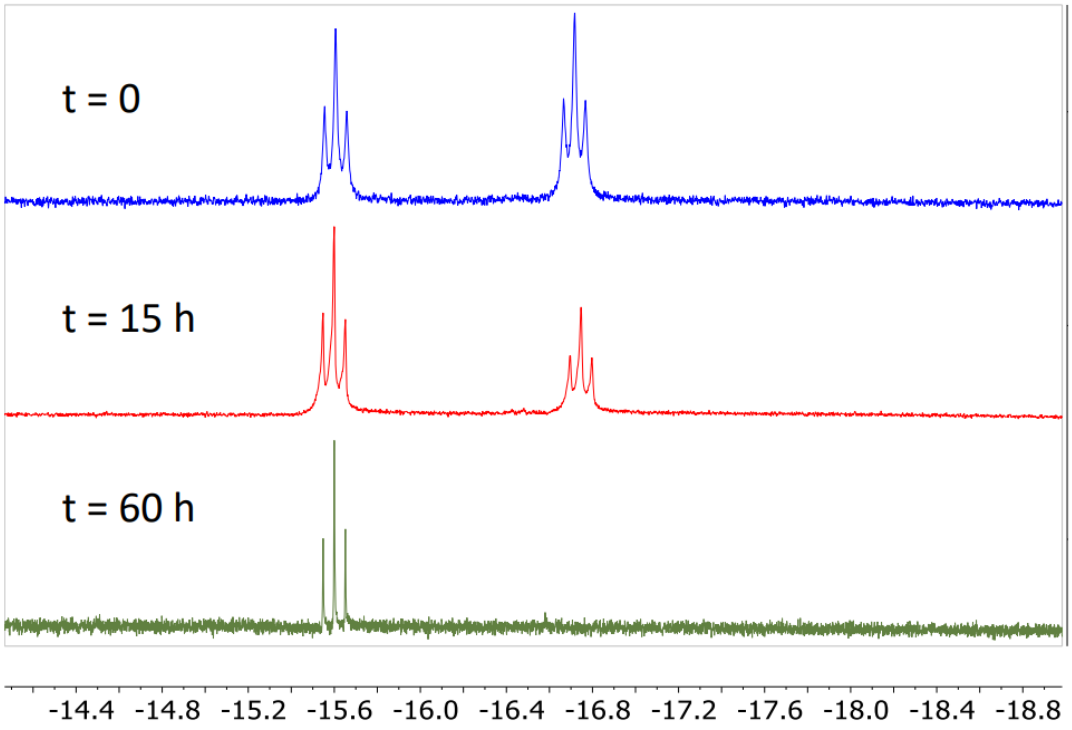

Figure S6: ^{1}H NMR spectrum in $CDCl_3$ for a period of 60 h at room temperature. The spectra of **2** reveal the occurrence of two distinct diastereoisomers.

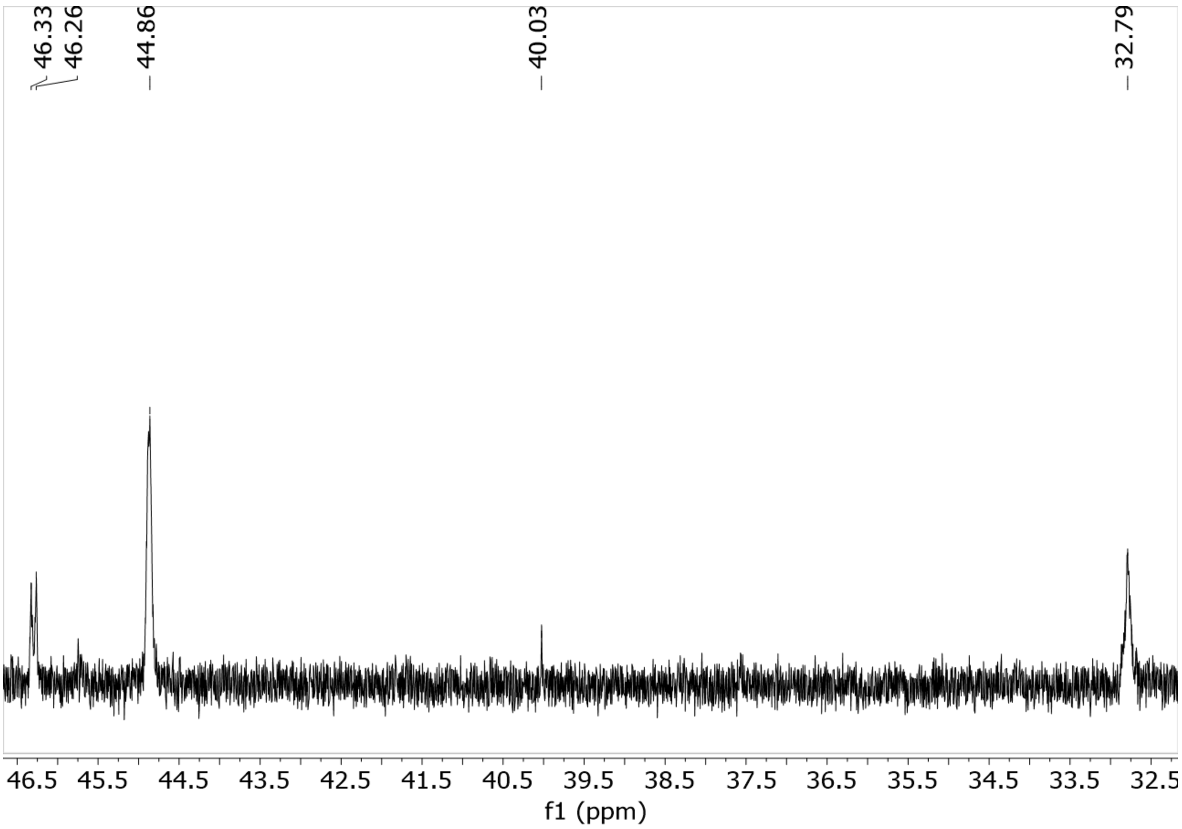

Figure S7: ^{31}P NMR spectrum in $CDCl_3$ of the kinetic mixture. Five species are observed.

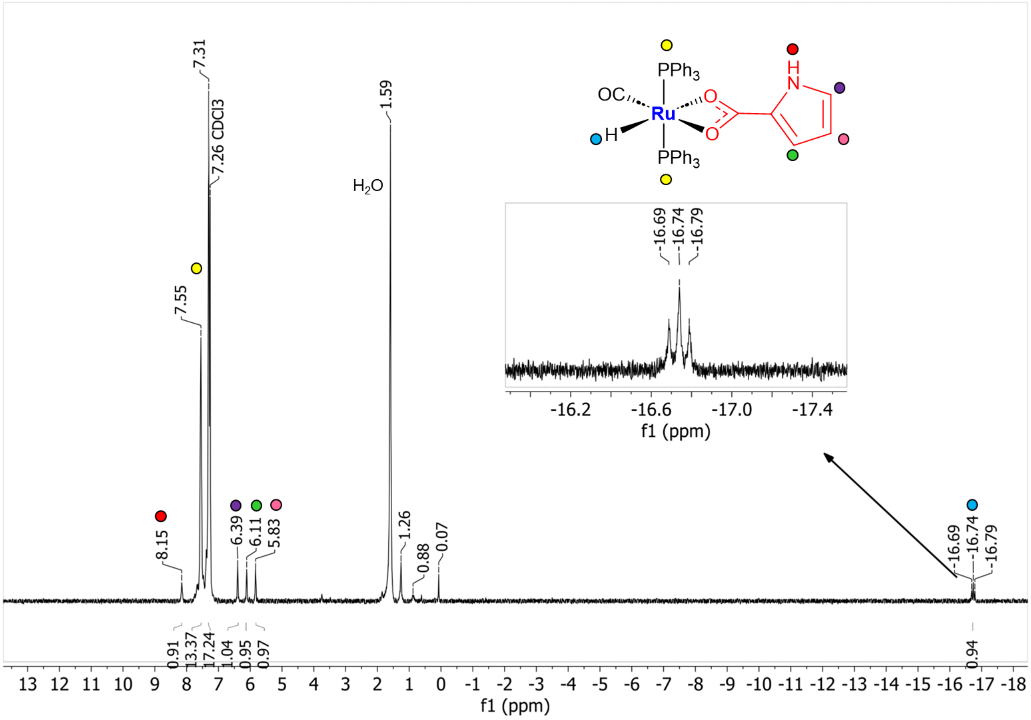

Figure S8: ^{1}H NMR spectrum of **2** in $CDCl_3$.

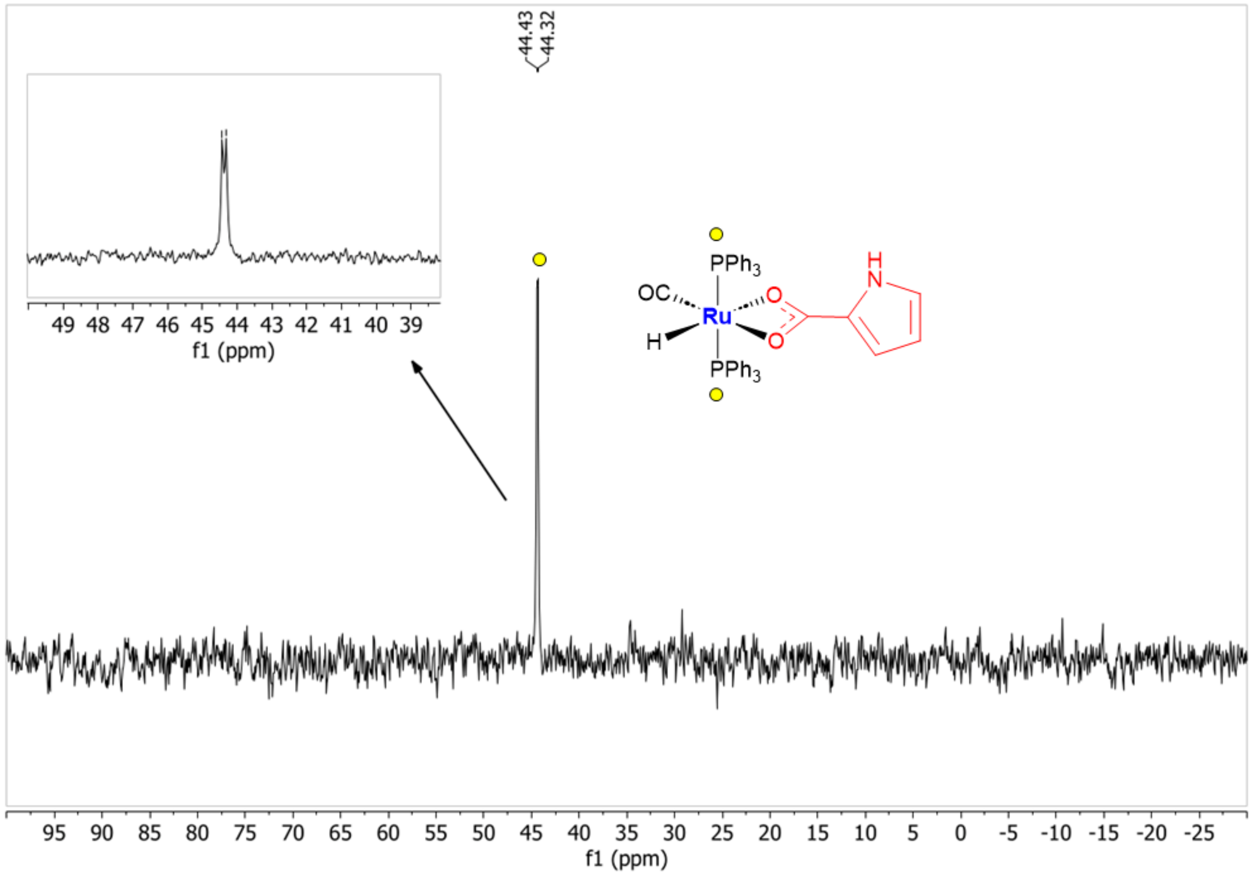

Figure S9: $^{31}P\{^{1}H\}$ NMR spectrum of **2** in $CDCl_3$.

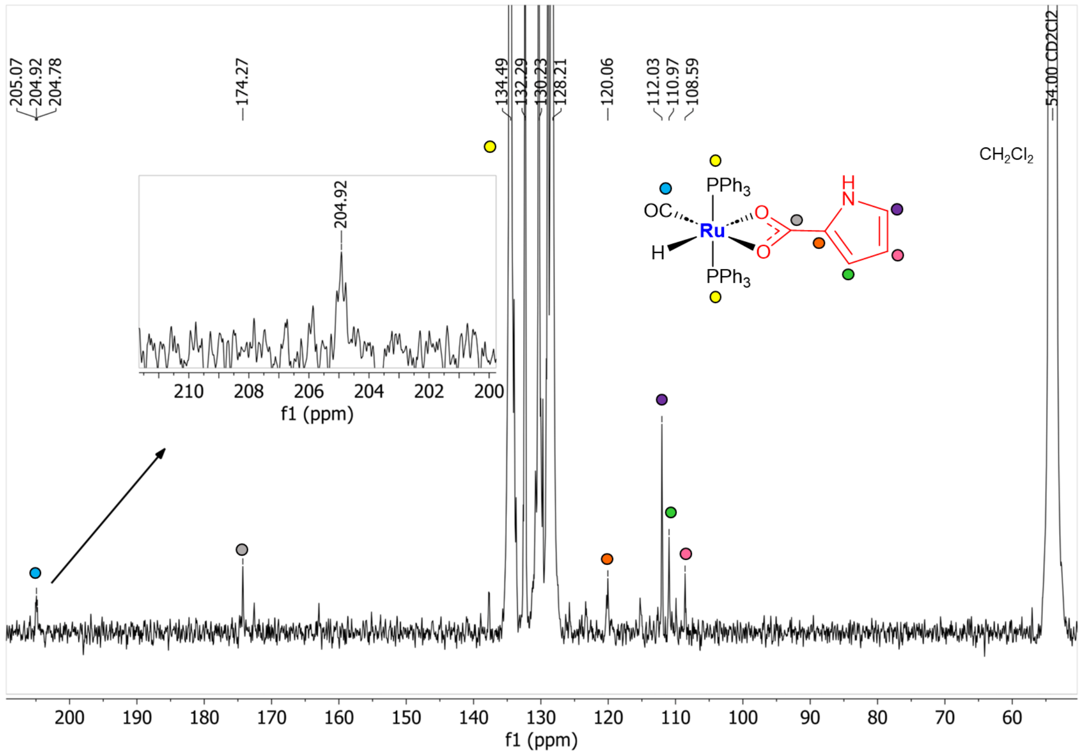

Figure S10: $^{13}C\{^{1}H\}$ NMR spectrum of **2** in $CDCl_2$.

Characterization of 3

Mass spectra of 3

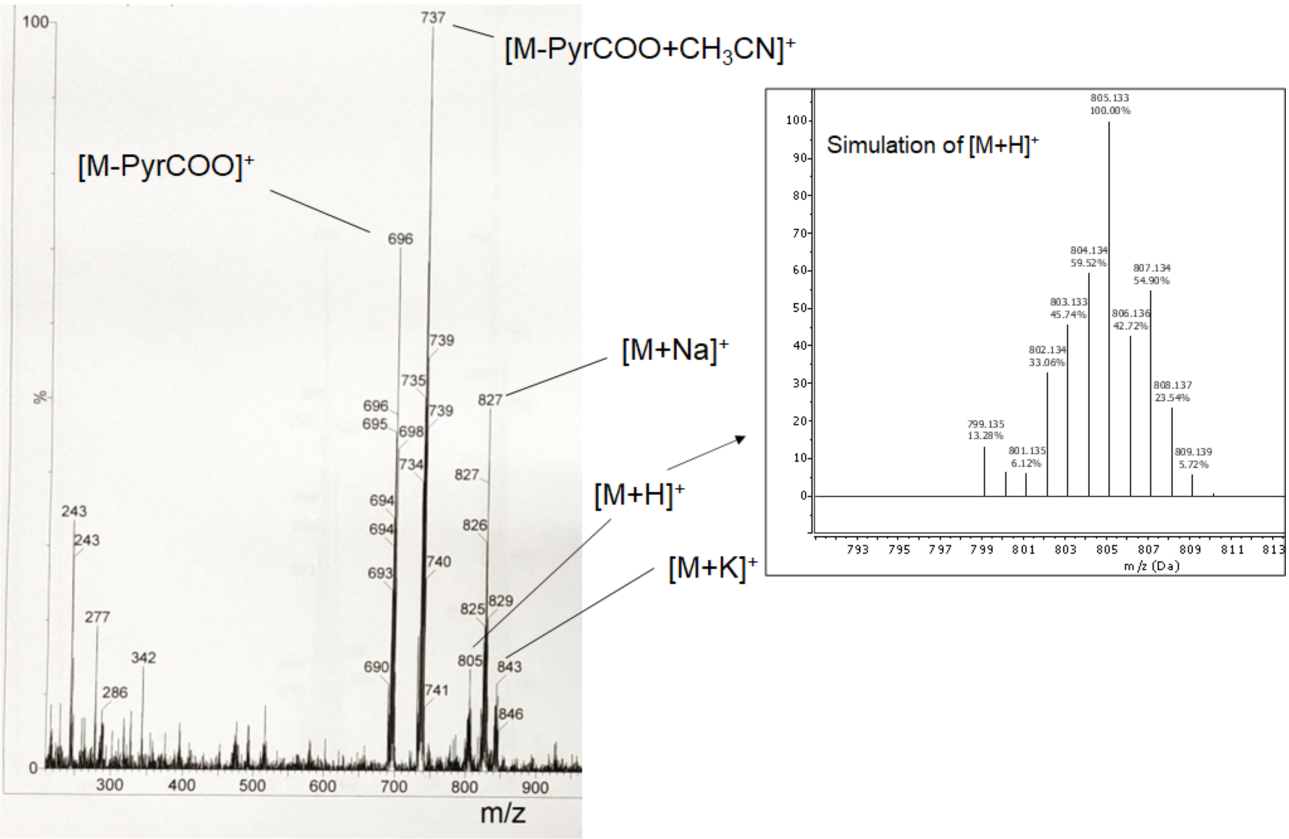

Figure S11: ESI-Mass Spectrum of **3** (positive mode, m/z: 200 – 950) in CH_3CN.

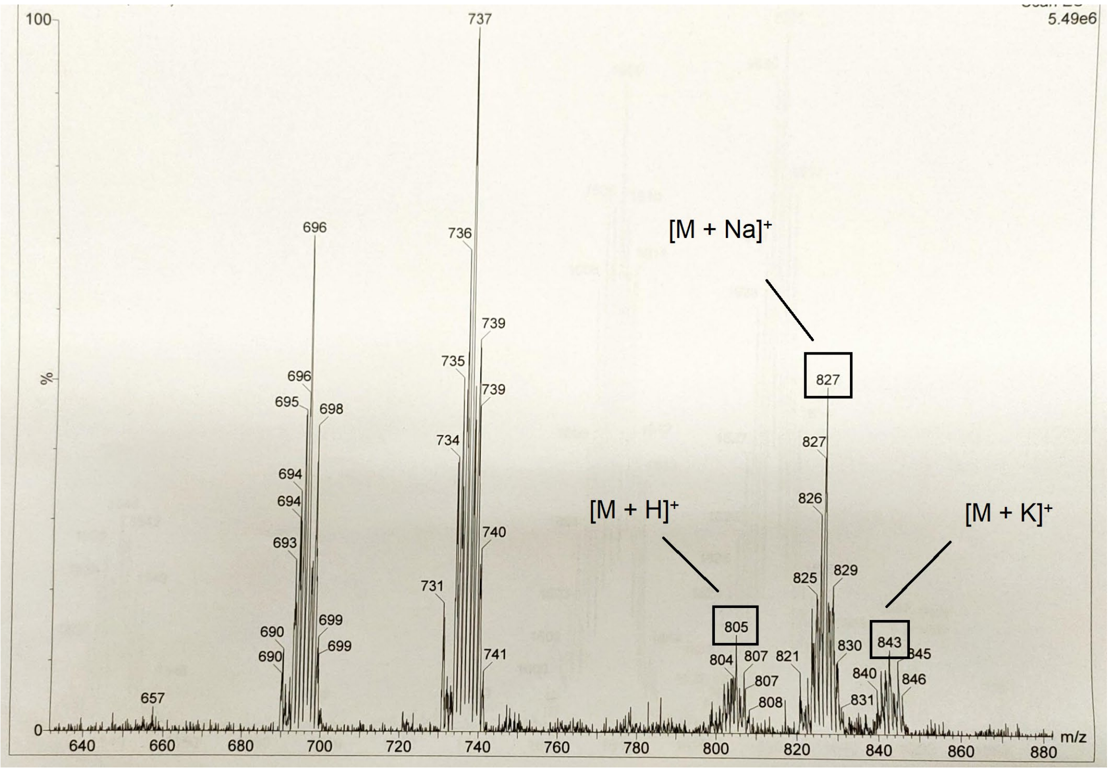

Figure S12: ESI-Mass spectrum of **3** (positive mode, m/z: 640 – 880) in CH_3CN.

IR spectrum of 3

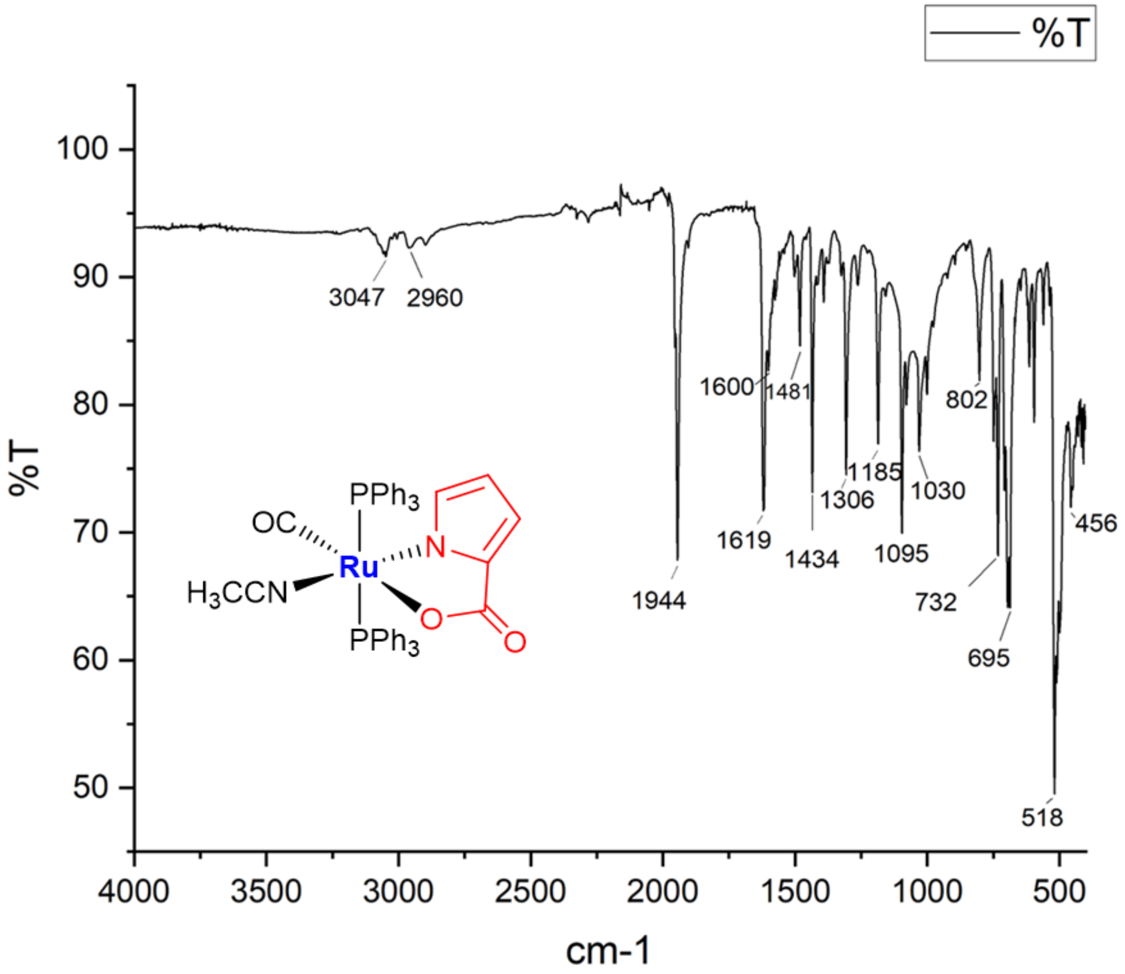

Figure S13: IR spectrum of **3** (ATR).

NMR spectra of **3**

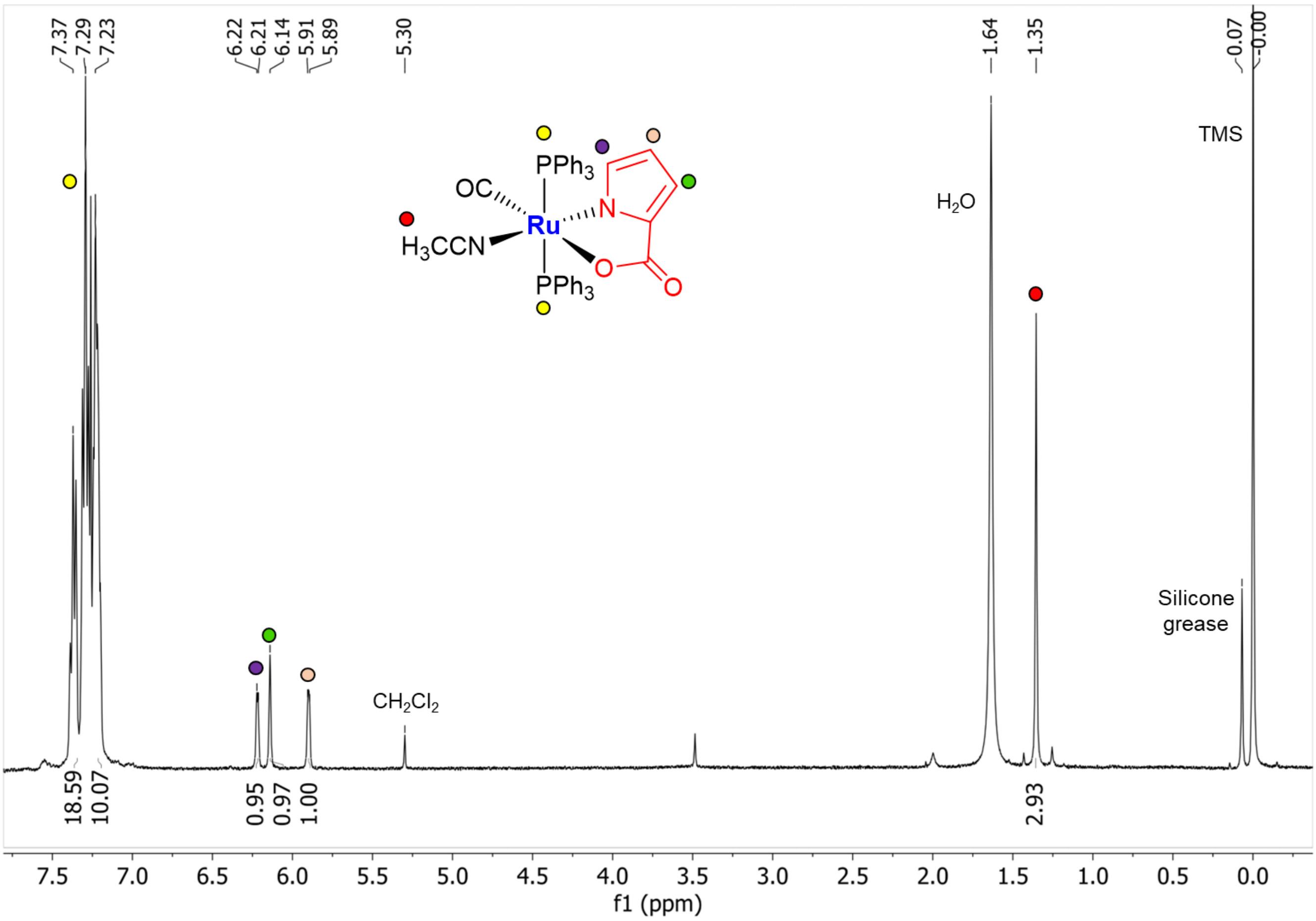

Figure S14: ^{1}H NMR spectrum of **3** in $CDCl_3$.

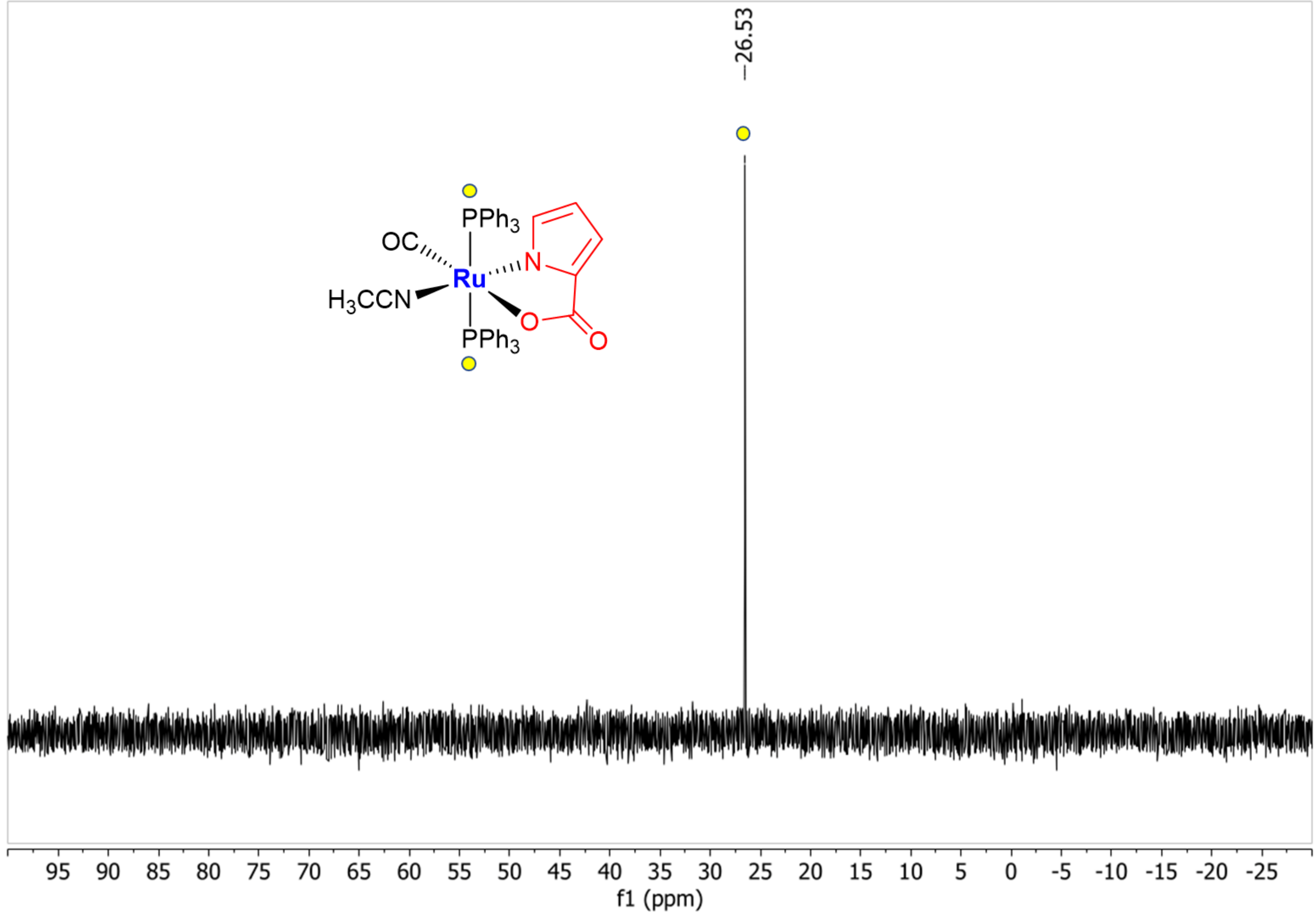

Figure S15: $^{31}P\{^{1}H\}$ NMR spectrum of **3** in $CDCl_3$.

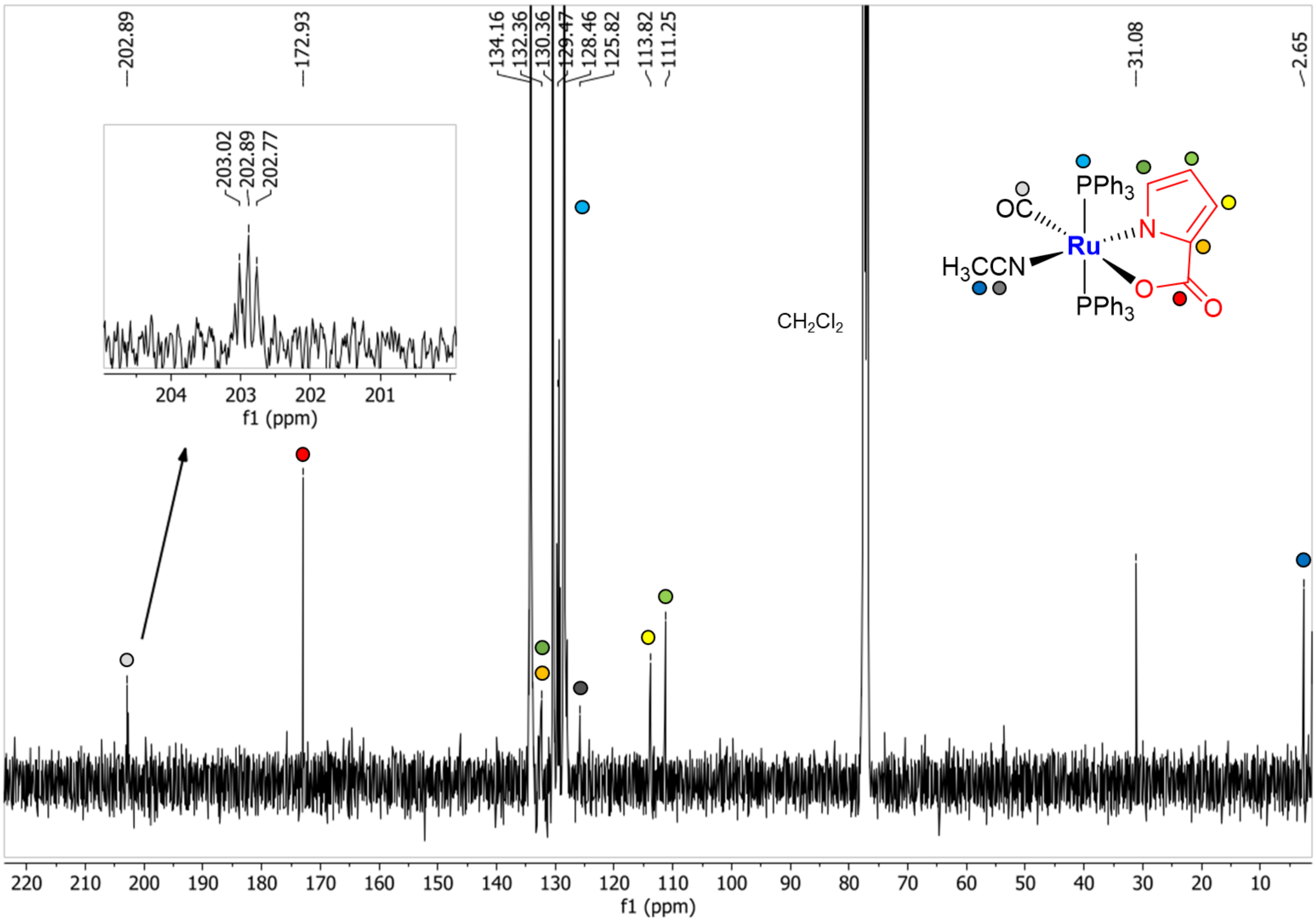

Figure S16: $^{13}C\{^{1}H\}$ NMR spectrum of **3** $CDCl_3$.

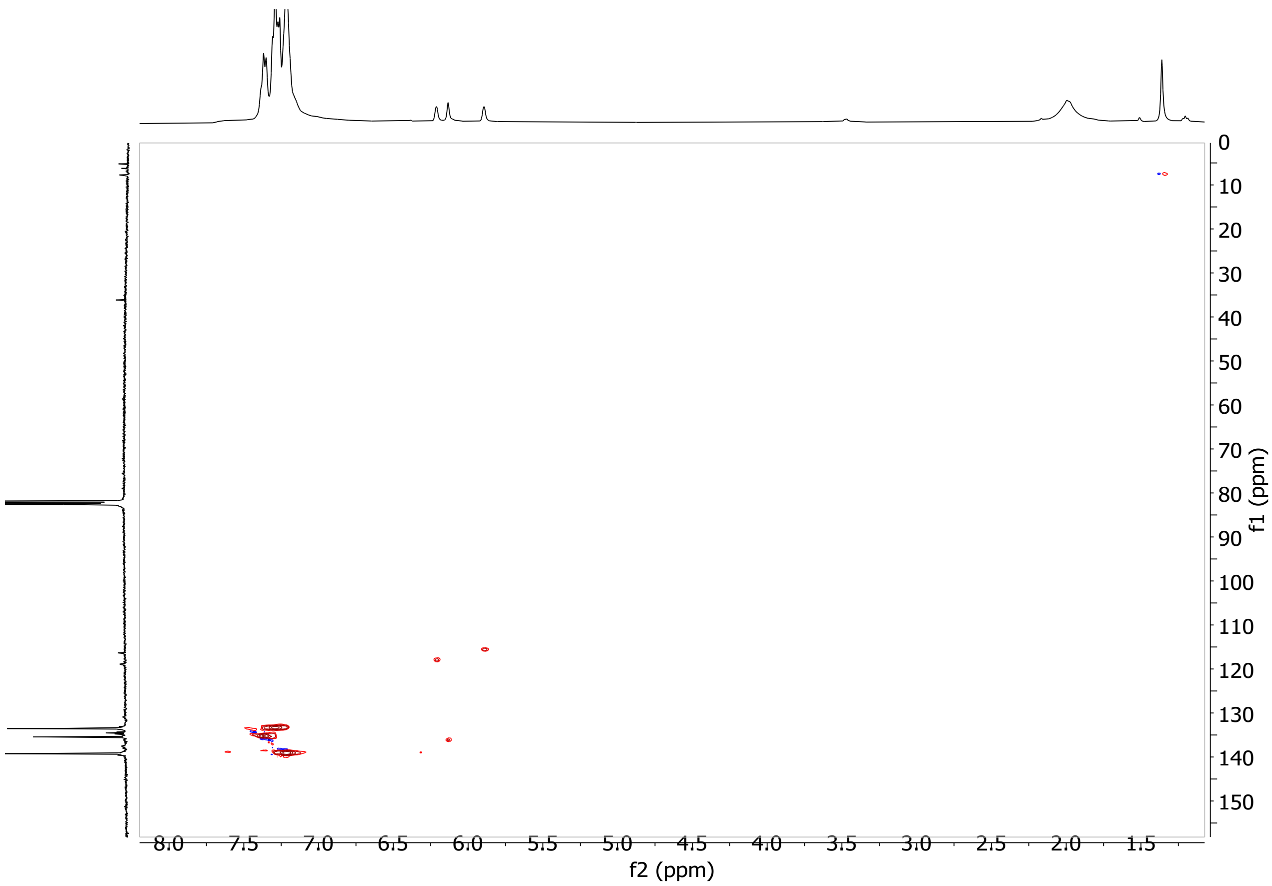

Figure S17: Heterocorrelated NMR spectrum {^{13}C,^{1}H} HSQC of **3** in $CDCl_3$.

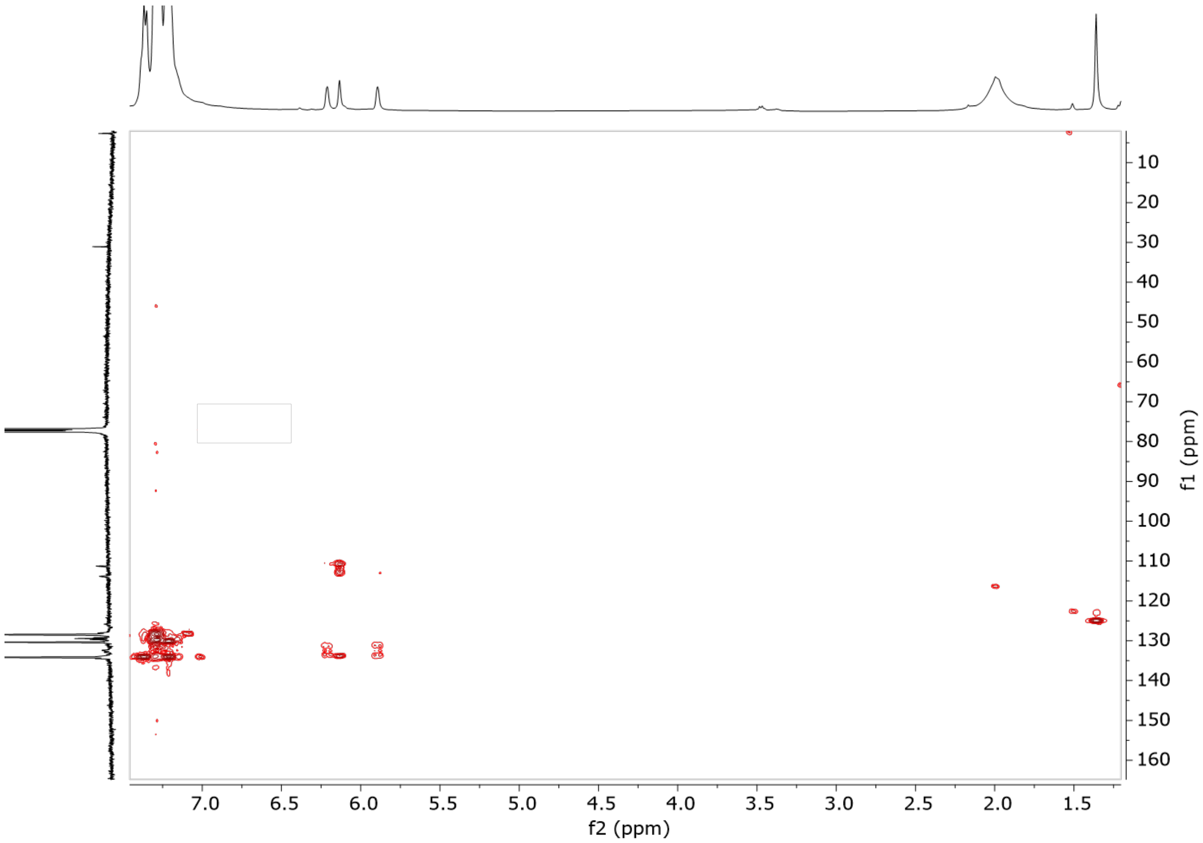

Figure S18: Heterocorrelated NMR spectrum {^{13}C,^{1}H} HMBC of **3** in $CDCl_3$.

Characterization of **4**

Mass spectrum of **4**

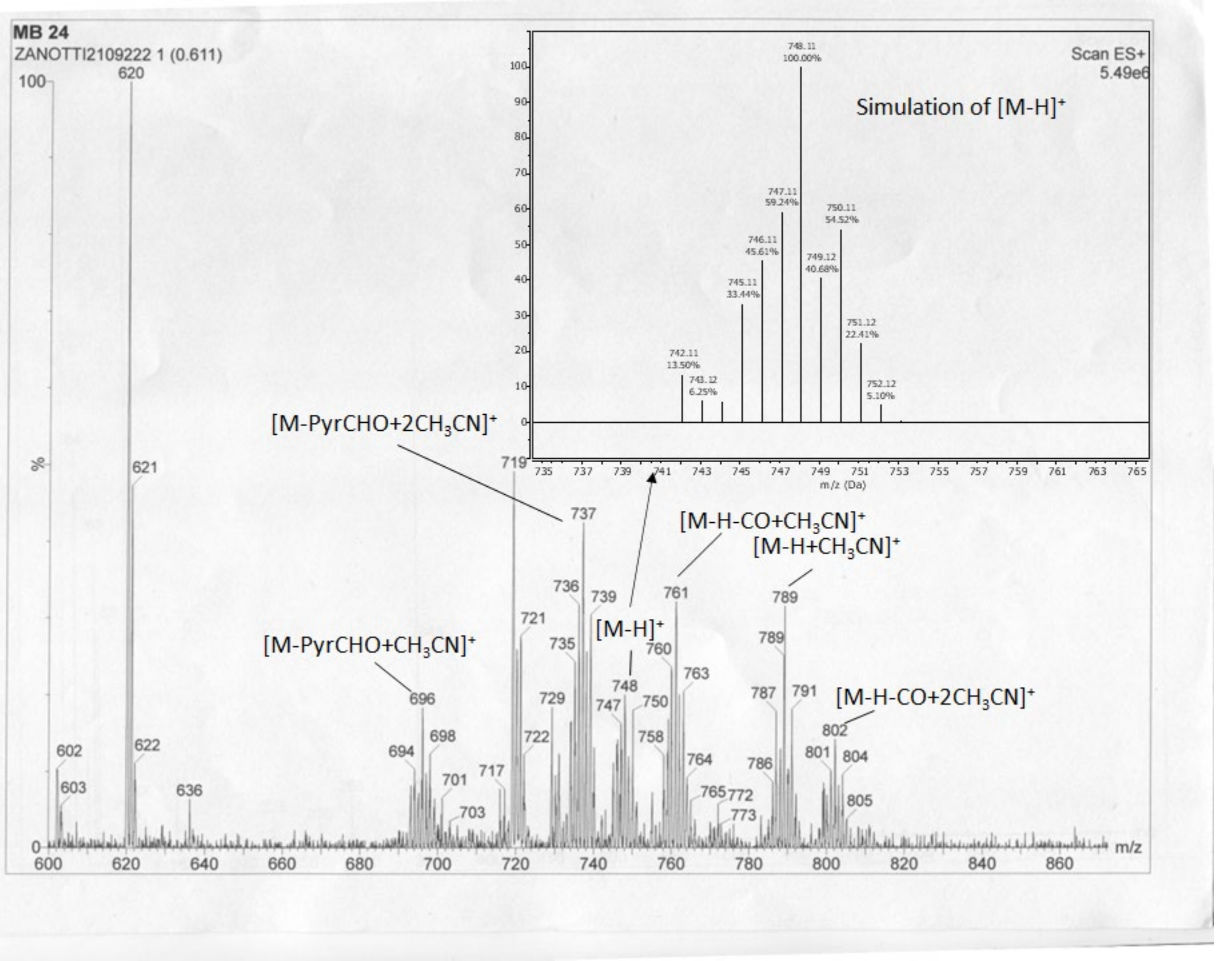

Figure S19: ESI-Mass spectrum of **4** (positive mode, m/z: 600 – 860) in CH_3CN compared to simulation.

IR spectrum of **4**

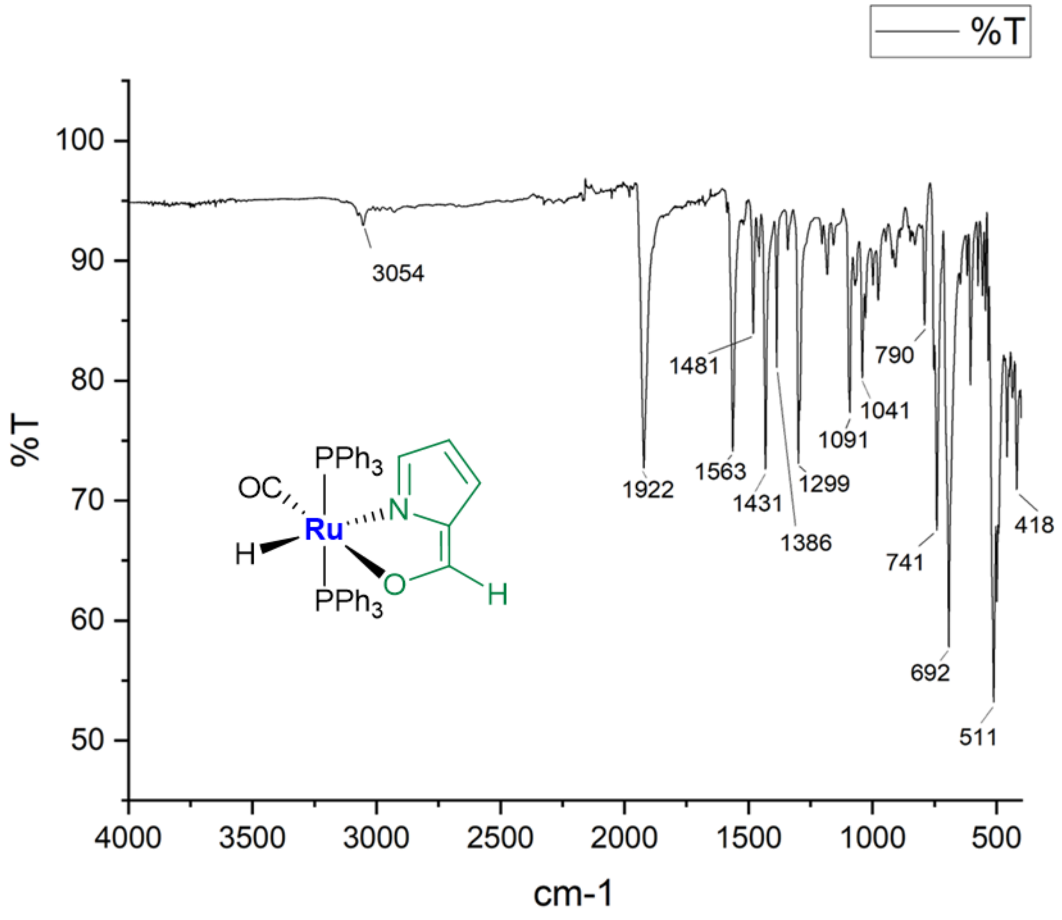

Figure S20: IR spectrum of **4** (ATR).

NMR spectra of **4**

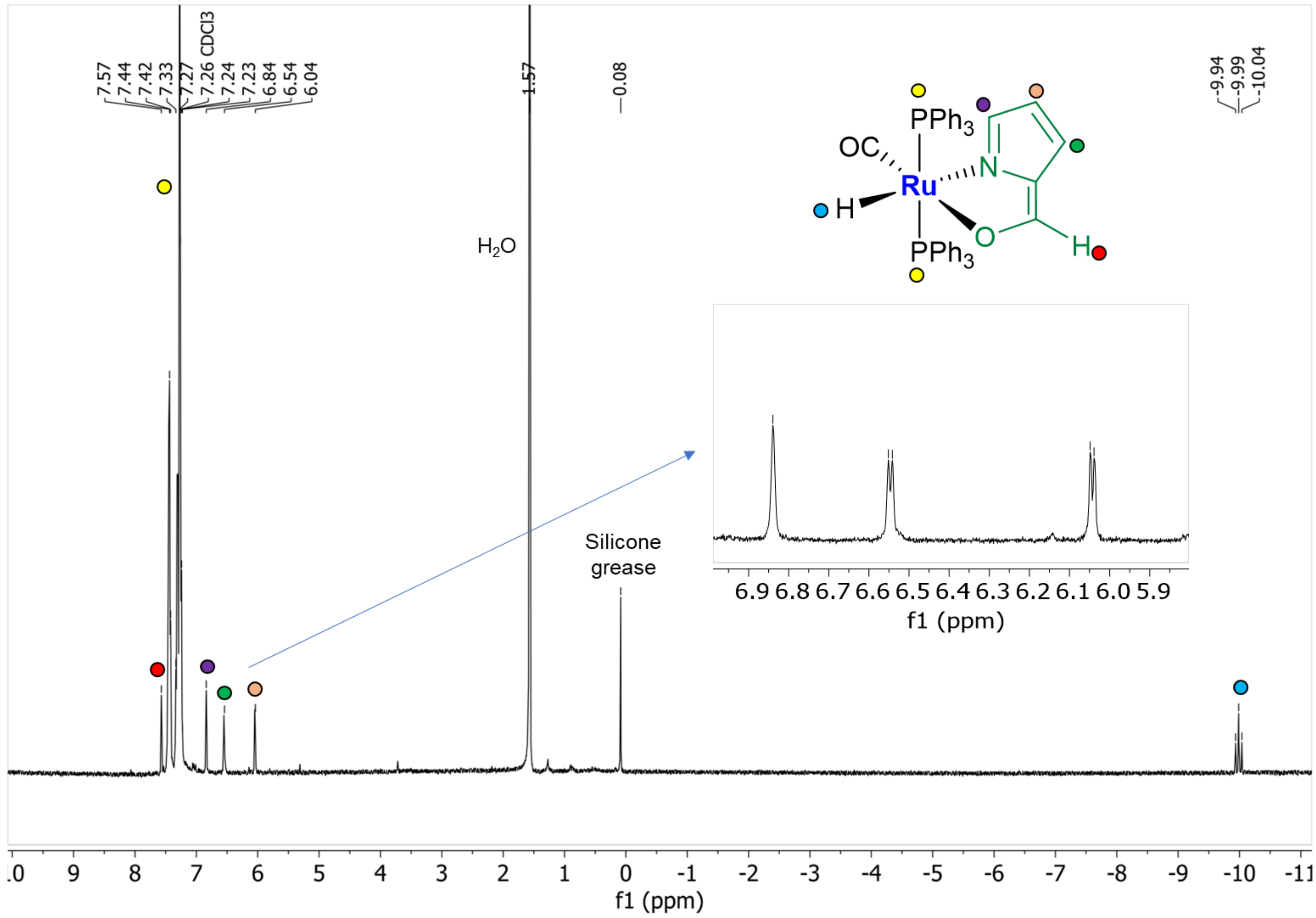

Figure S21: ^{1}H NMR spectrum of **4** in $CDCl_3$.

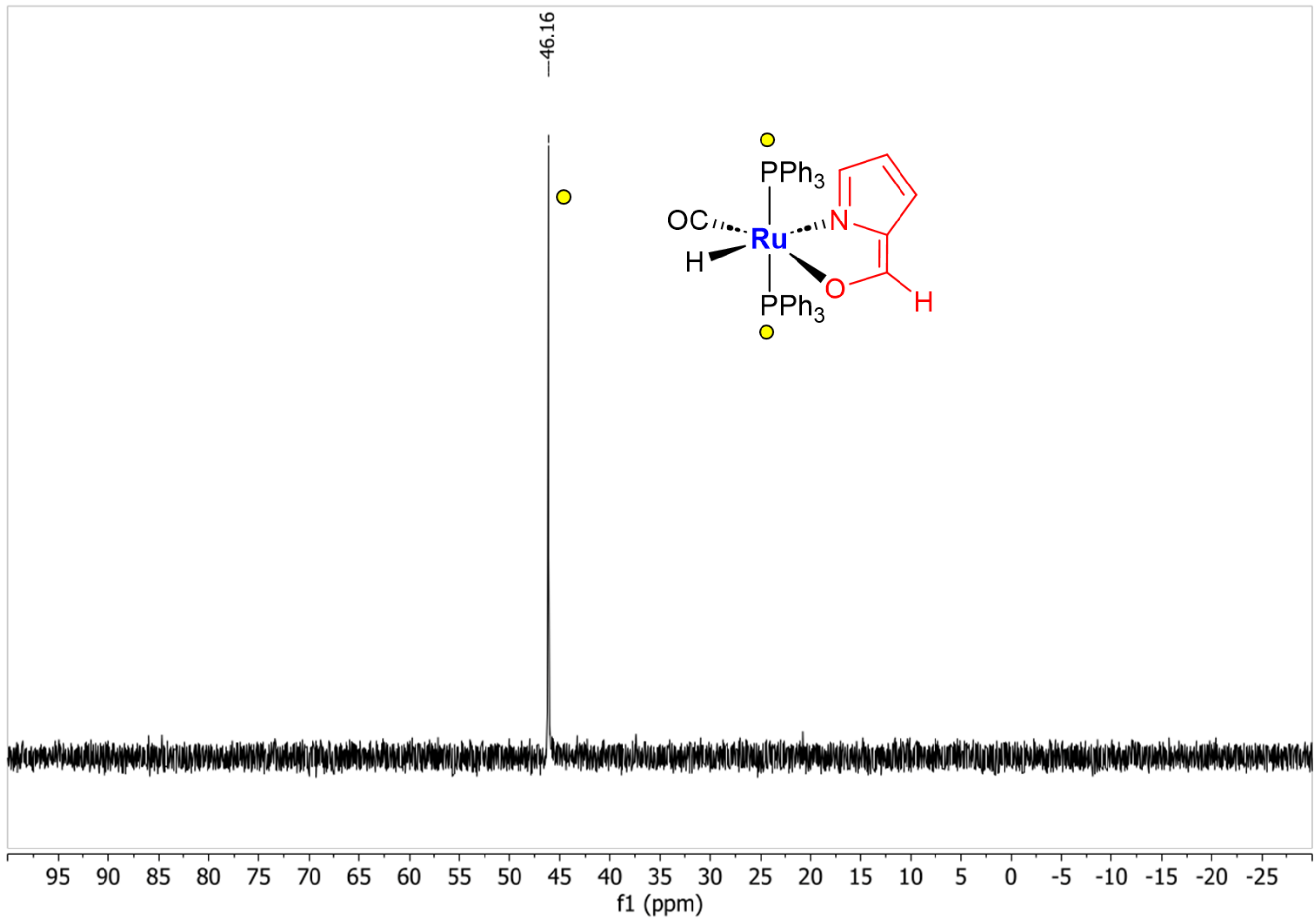

Figure S22: $^{31}P\{^{1}H\}$ NMR spectrum of **4** in $CDCl_3$.

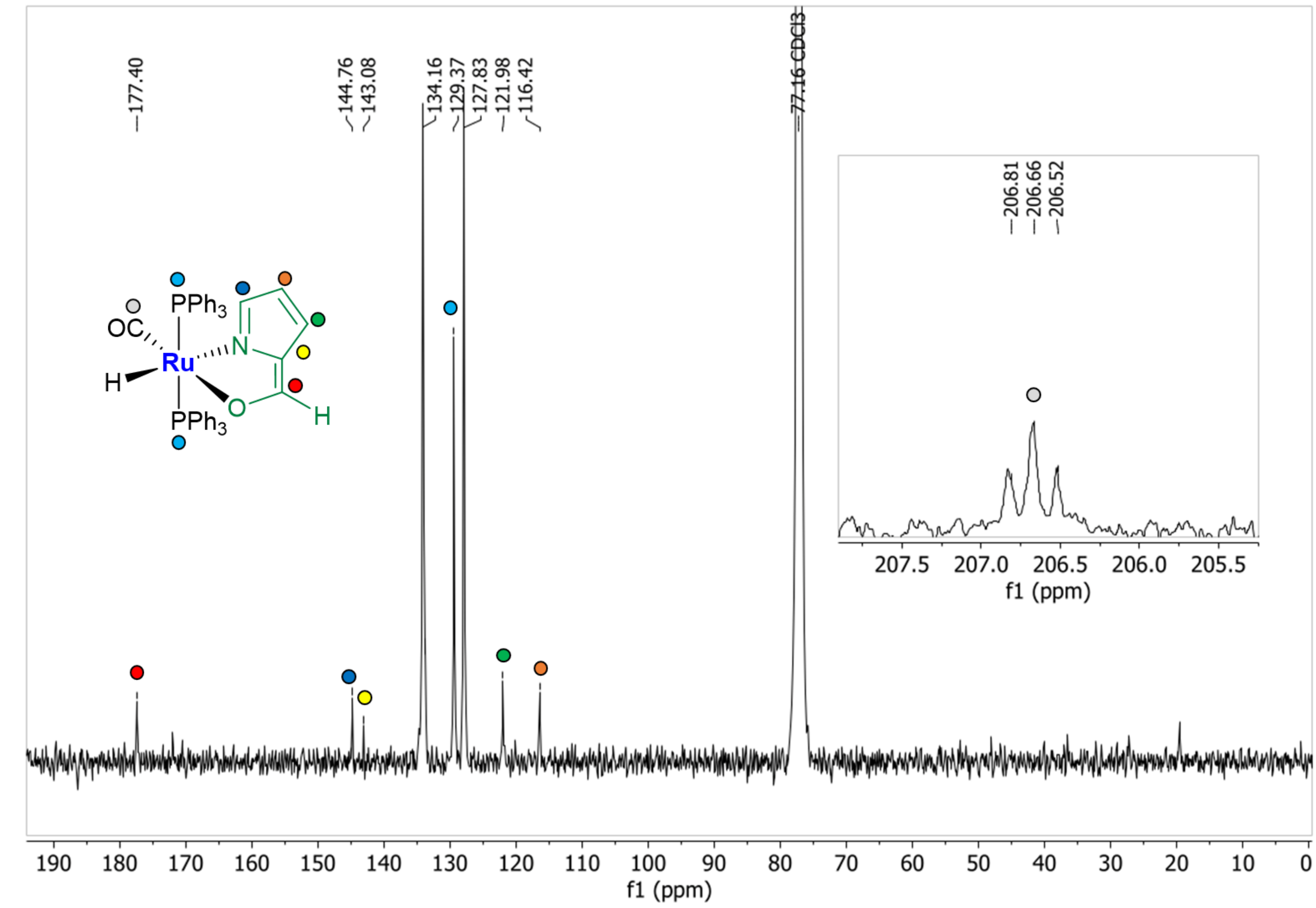

Figure S23: $^{13}C\{^{1}H\}$ NMR spectrum of **4** in $CDCl_3$.

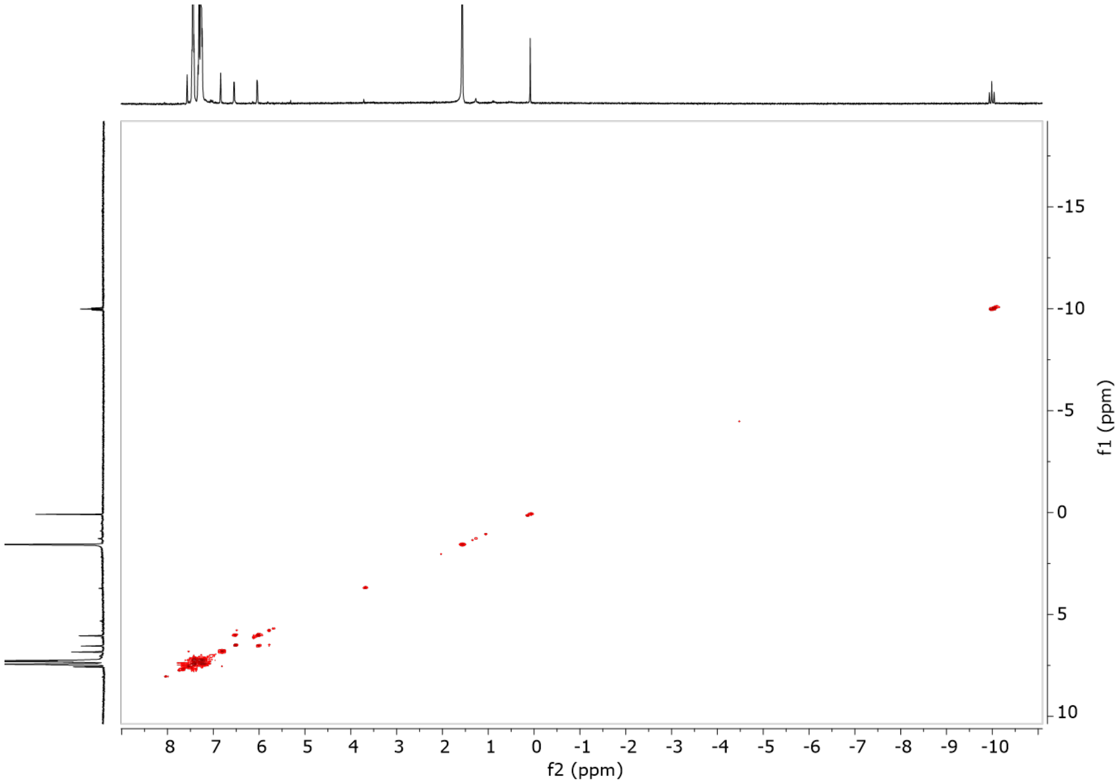

Figure S24: Homocorrelated $\{^{1}H\}$ COSY spectrum of **4** in $CDCl_3$.

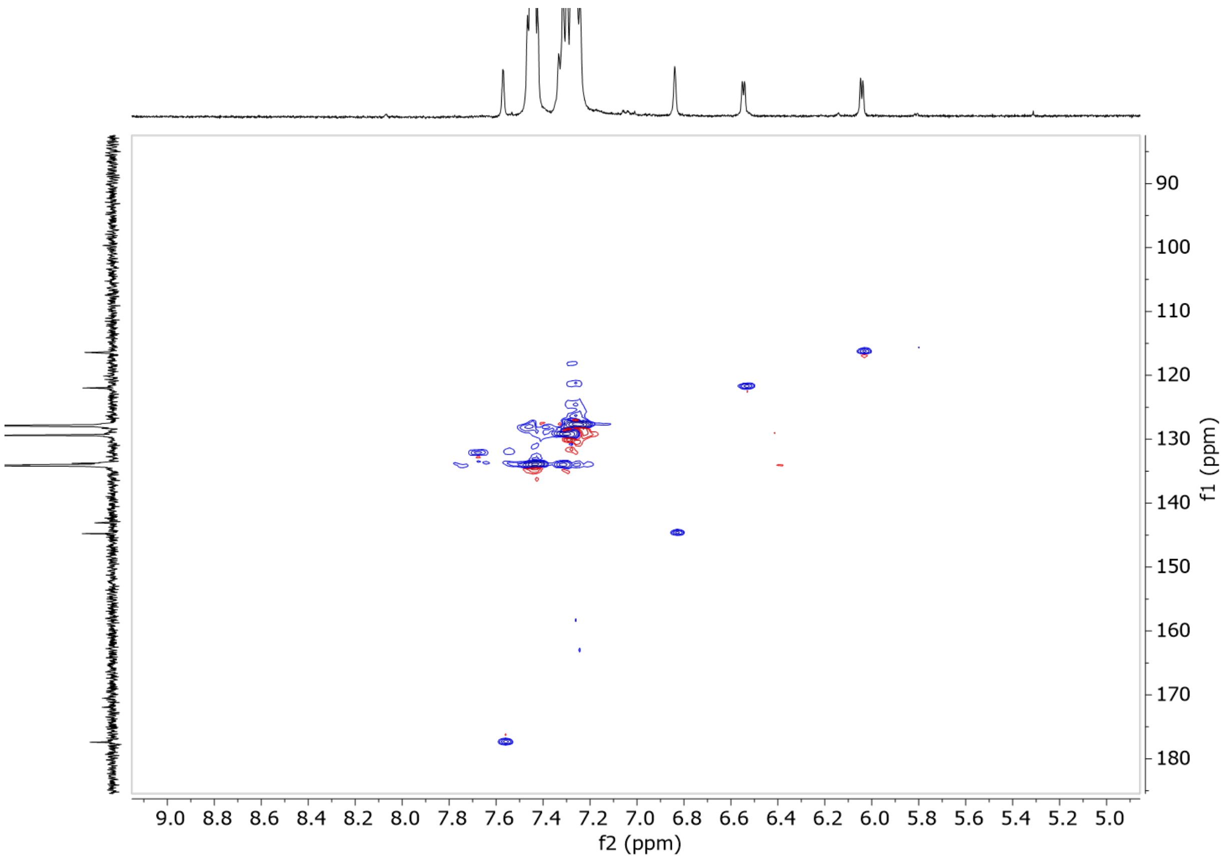

Figure S25: Heterocorrelated NMR spectrum {^{13}C,^{1}H} HSQC of **4** in $CDCl_3$.

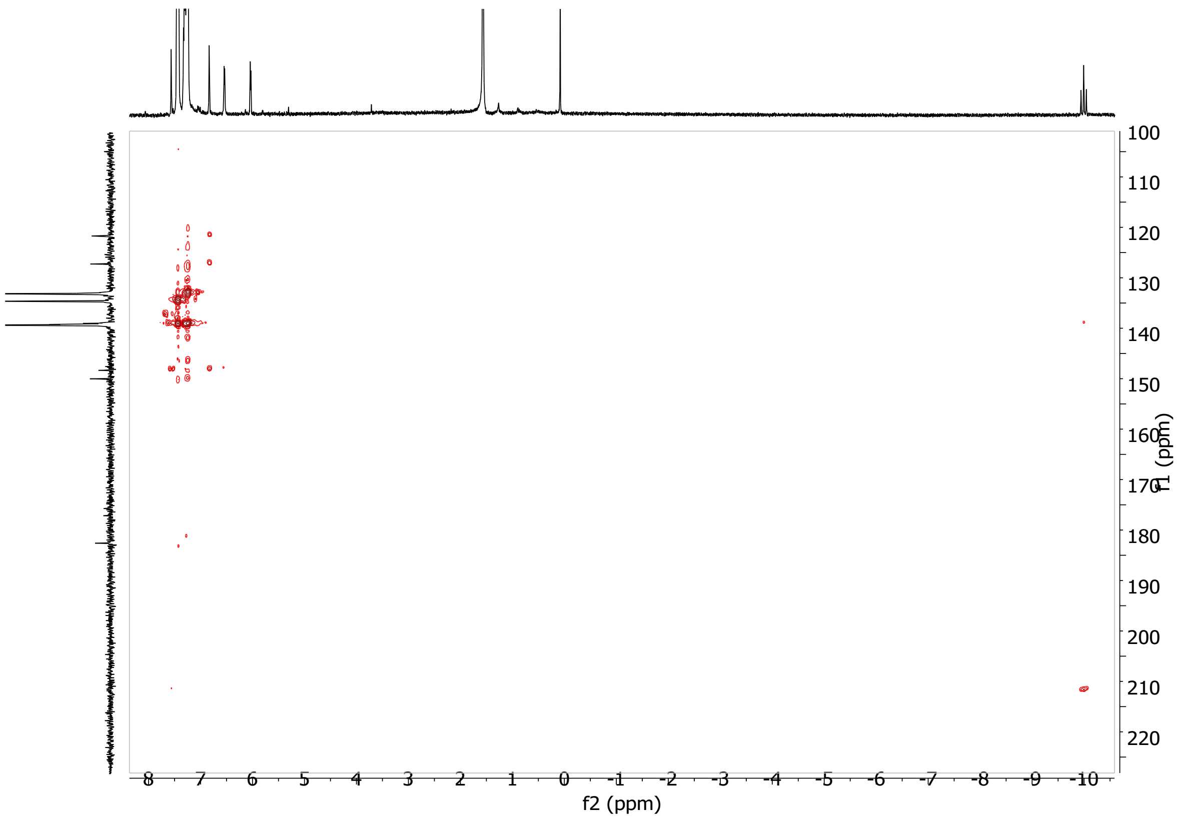

Figure S26: Heterocorrelated NMR spectrum {^{13}C,^{1}H} HMBC of **4** in $CDCl_3$.

Characterization of **5**

Mass Spectrum of **5**

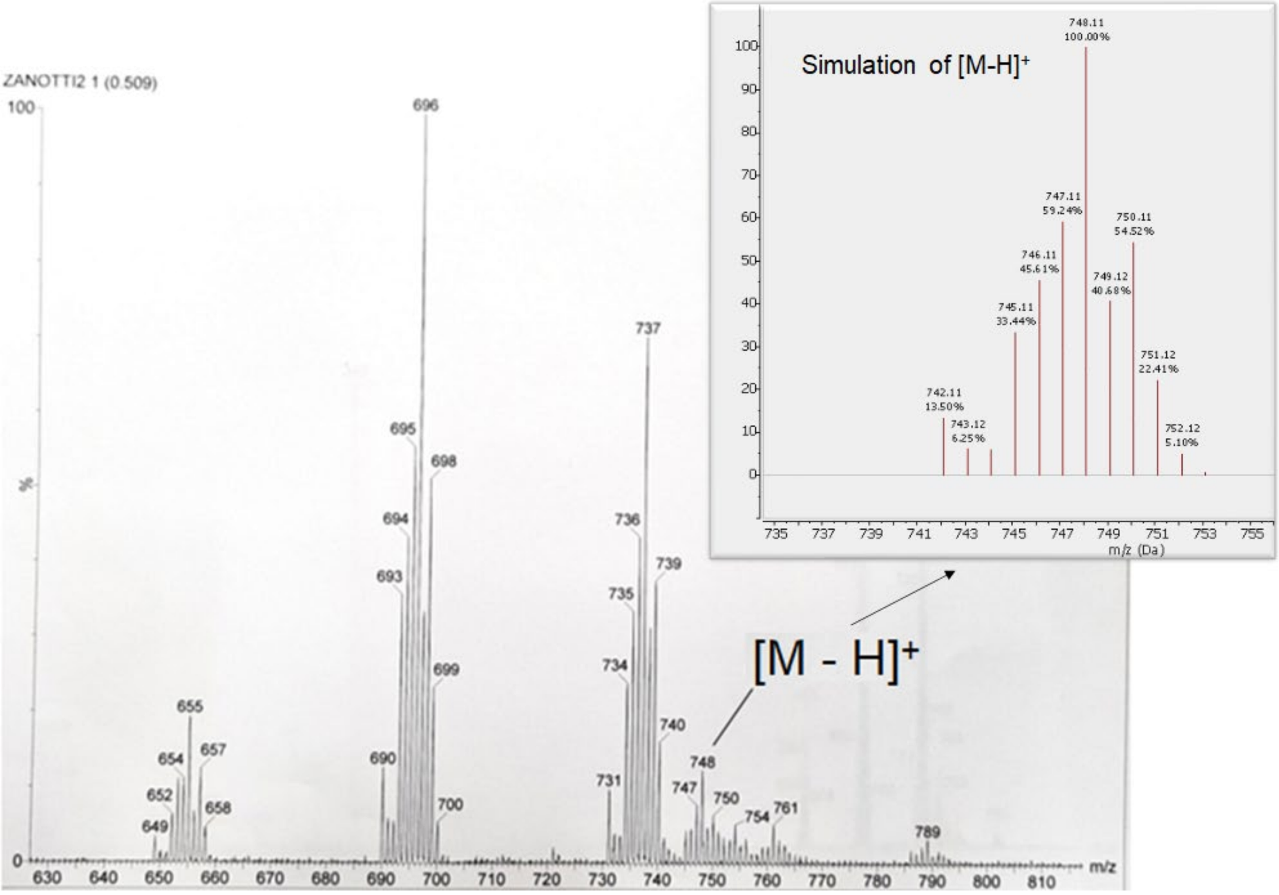

Figure S27: ESI-Mass spectrum of **5** (positive mode, m/z: 630 – 810) in CH_3CN.

IR spectrum of 5

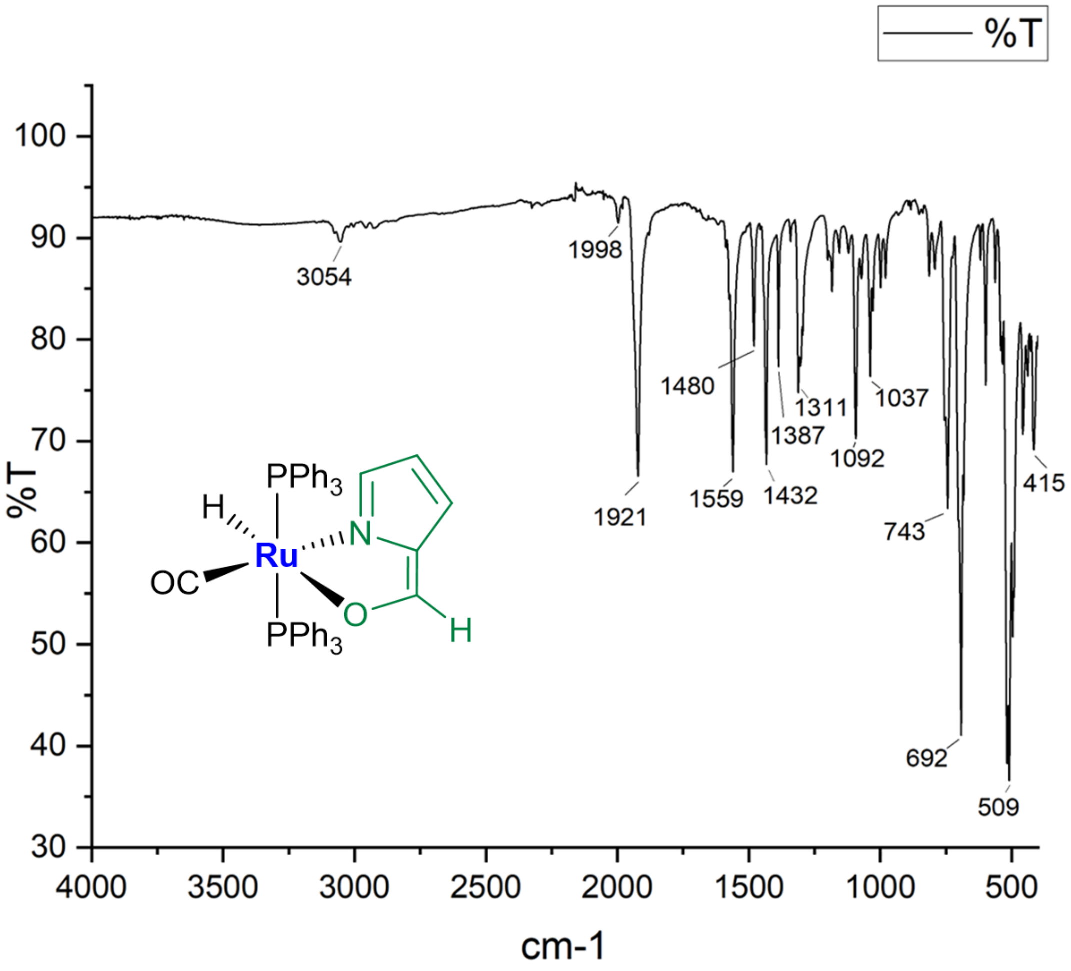

Figure S28: IR spectrum of **5** (ATR).

NMR spectra of 5

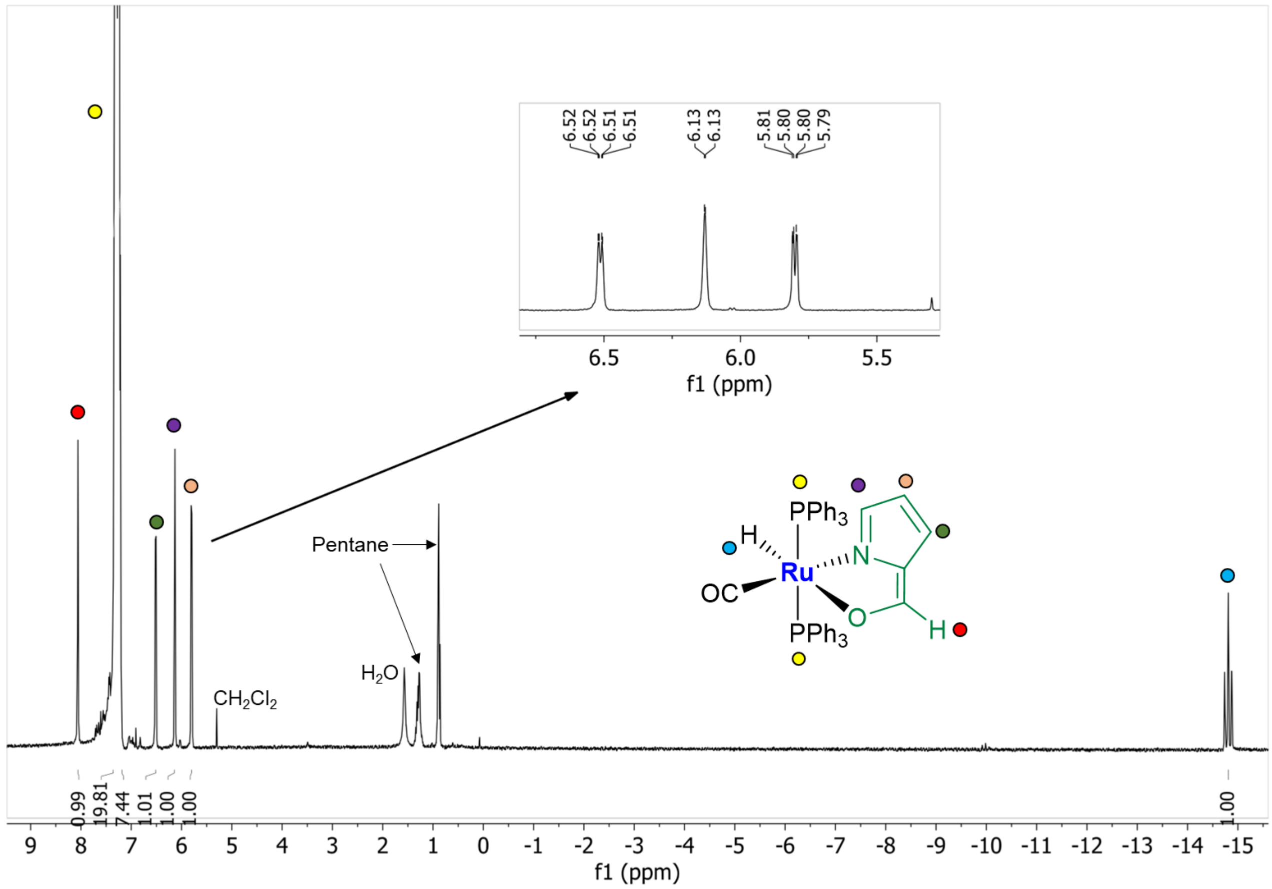

Figure S29: ^{1}H NMR spectrum of **5** in $CDCl_3$.

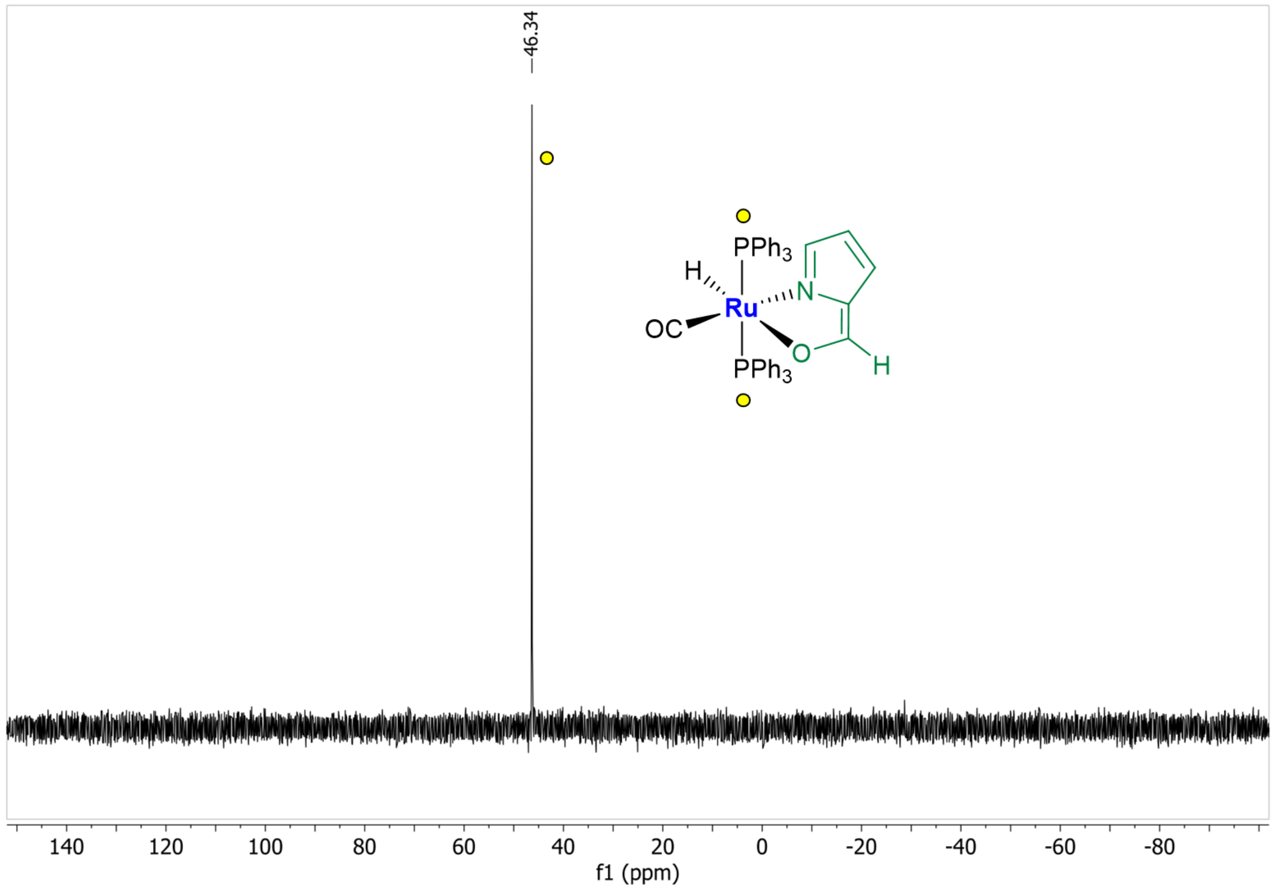

Figure S30: $^{31}P\{^{1}H\}$ NMR spectra of **5** in $CDCl_3$.

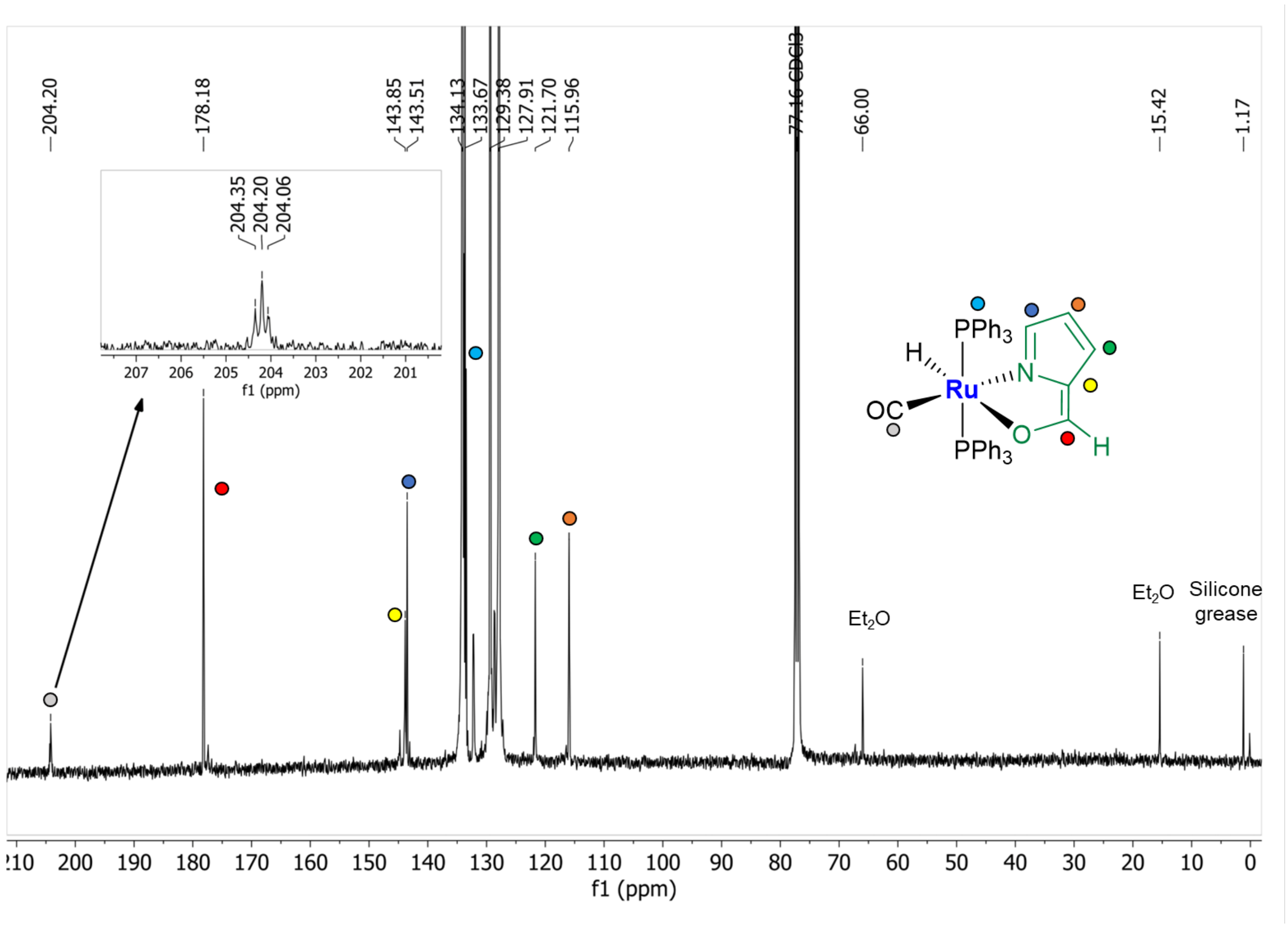

Figure S31: $^{13}C\{^1H\}$ NMR spectrum of **5** in $CDCl_3$.

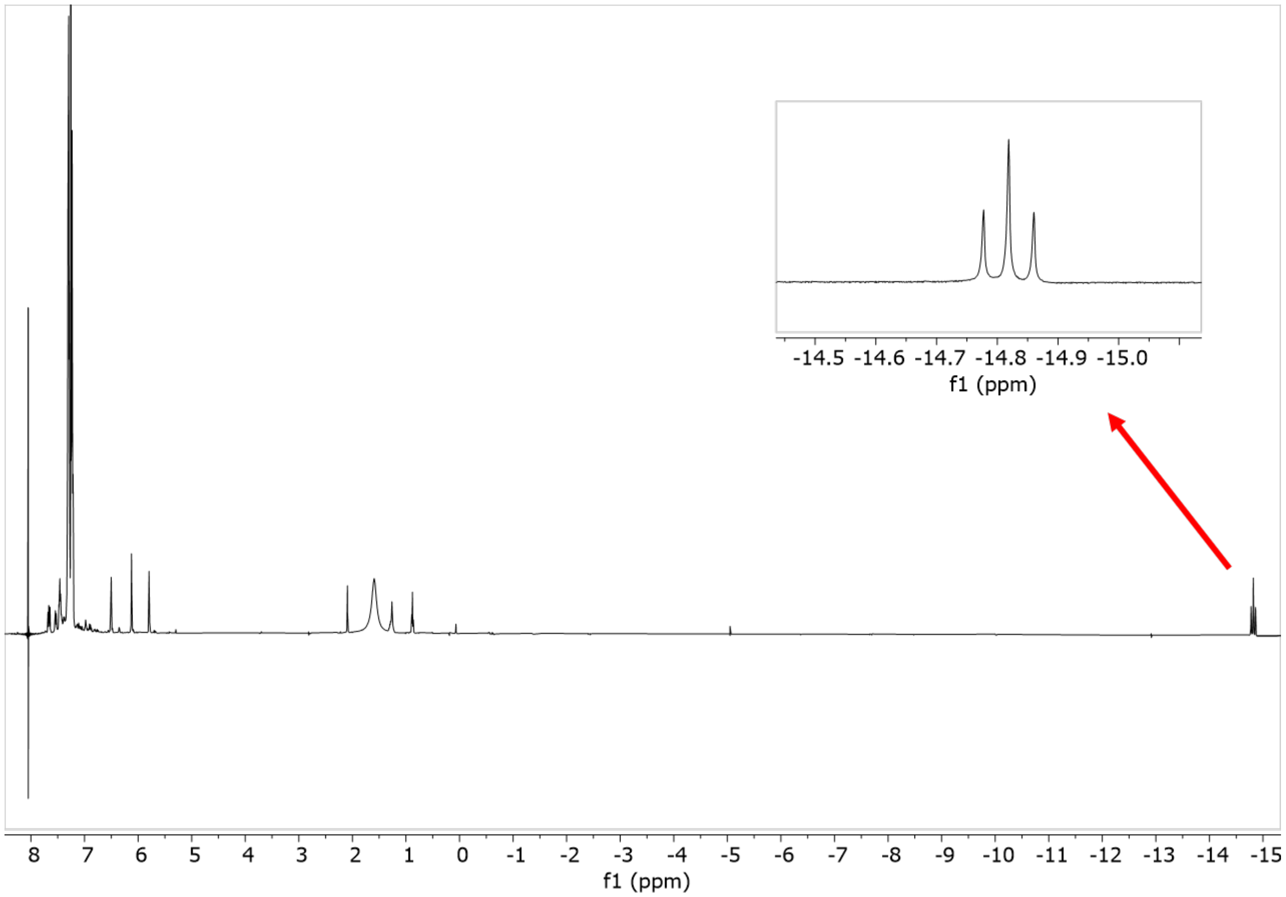

Figure S32: [1]H Homonuclear decoupling: upon the irradiation of the CHO aldehyde proton, the molteplicity of the Ru-hydride signal converts from double triplet to triplet.

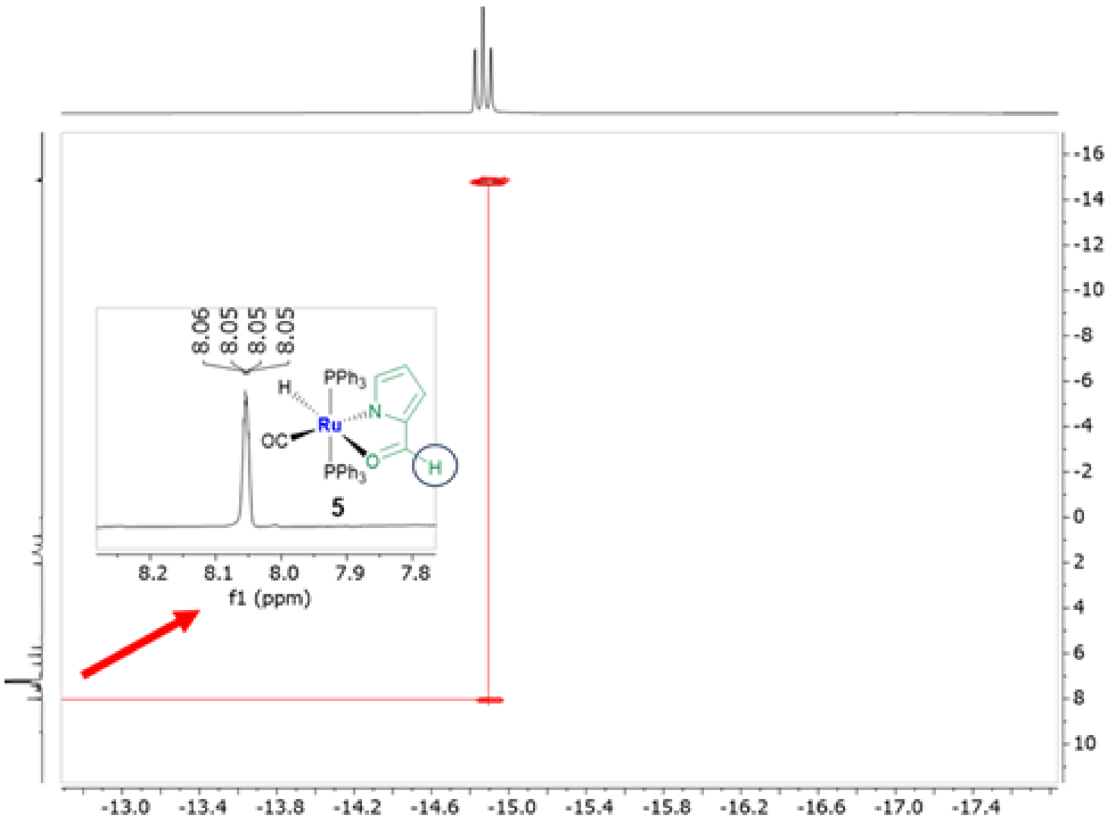

Figure S33: 2D COSY NMR spectrum of **5**. The coupling between the hydride triplet and the multiplet of the CHO group is observed.

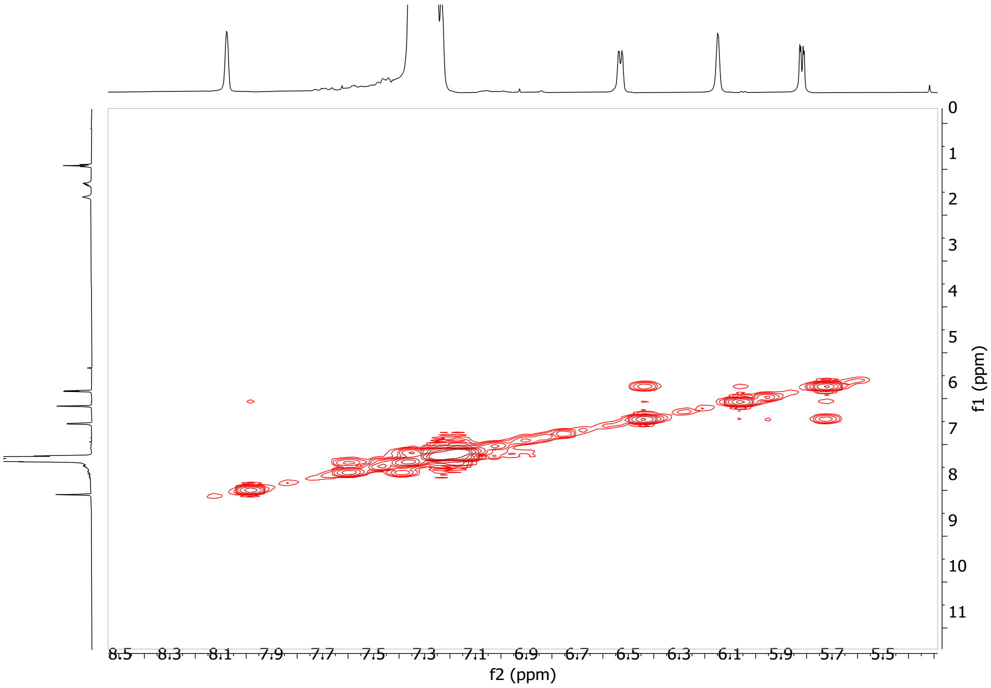

Figure S34: Homocorrelated NMR spectrum {^{1}H} COSY of **5** in $CDCl_3$.

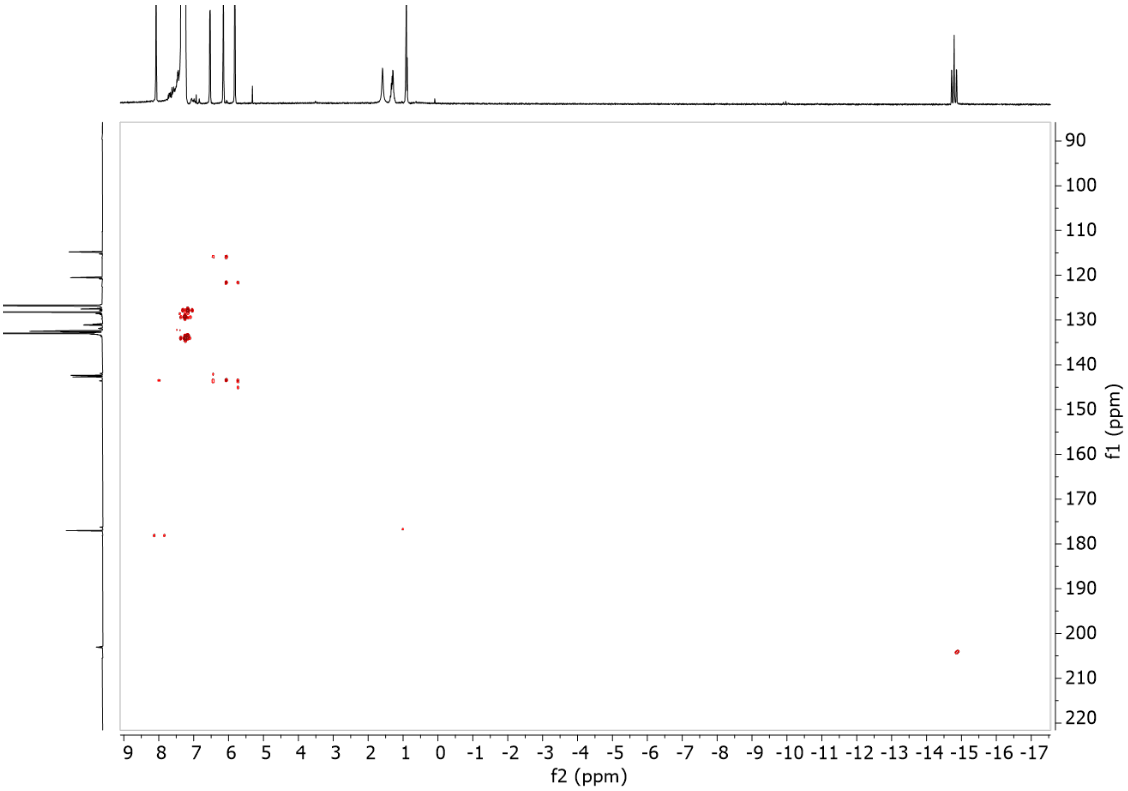

Figure S35: Heterocorrelated NMR spectrum {^{13}C,^{1}H} HMBC of **5** in $CDCl_3$.

DFT CALCULATIONS

Mayer Bond Orders

MBO complex **2**

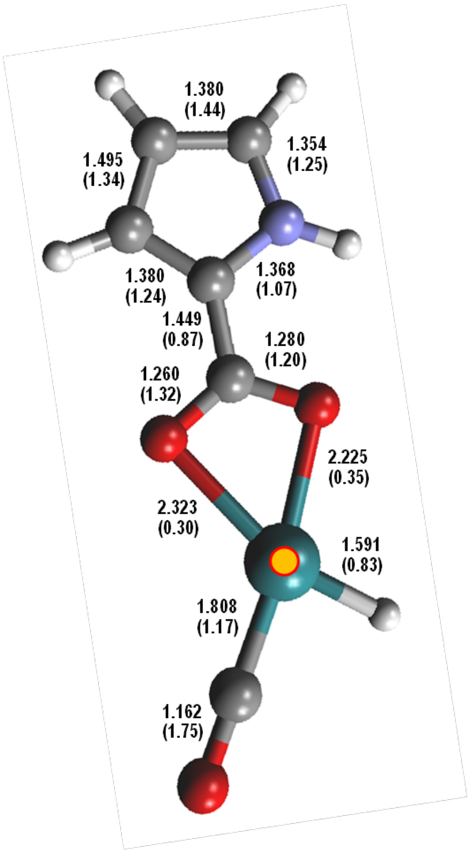

Figure S36: Calculated bond lengths of **2**. Mayer Bond Orders are reported in brackets.

Table S1. Summarized dipole bindings of **2**.

Atoms	Distance (Å)
NH-C(Ph)	3.44
CH(Ph)-O(carboxylate)	2.60
CH(Ph)-O(carboxylate)	3.24
RuH-CH(Ph)	1.93
RuCO-CH(Ph)	1.95

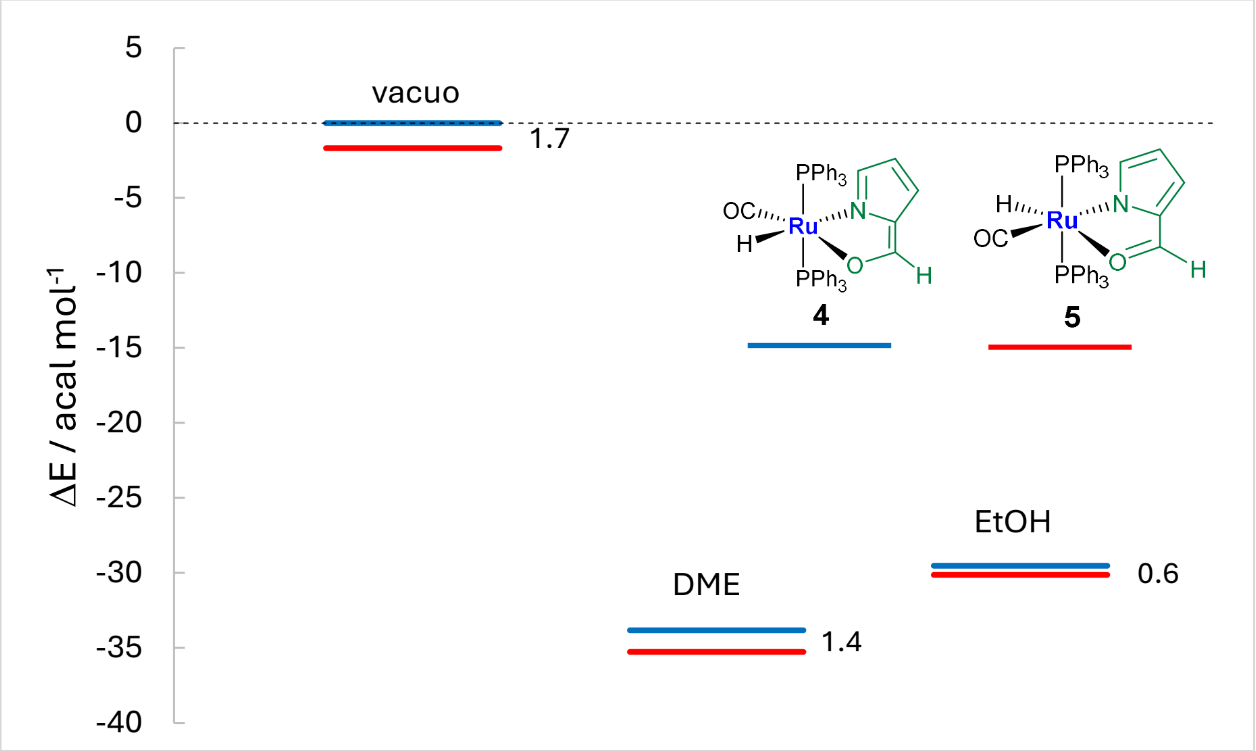

Fig S37: DFT calculations for **4** and **5** have been performed both in vacuo, 1,2-DME and EtOH solvents.

X-RAY DIFFRACTION STUDIES

Crystal Data

Table S2. Crystal data and structure refinement for **3-5**.

Compound	**3**	**4**	**5**
Formula	$C_{44}H_{36}N_2O_3P_2Ru$	$C_{42}H_{35}NO_2P_2Ru$	$C_{42}H_{35}NO_2P_2Ru$
Fw	803.76	748.72	748.72
T, K	100	100	100
λ, Å	1.54178	0.71073	1.54178
Crystal symmetry	Monoclinic	Monoclinic	Monoclinic
Space group	$P2_1/n$	$P2_1/c$	$C2/c$
a, Å	13.7700(4)	13.1063(8)	16.7723(14)
b, Å	9.5479(3)	14.9846(6)	10.7193(10)
c, Å	27.9662(8)	18.8647(9)	19.5464(13)
α	90	90	90
β	95.449(2)	108.890(6)	101.826(4)
γ	90	90	90
Cell volume, $Å^3$	3660.23(19)	3505.3(3)	3439.6(5)
Z	4	4	4
D_C, Mg m^{-3}	1.459	1.419	1.446
Absorption coefficient, mm^{-1}	4.644	0.576	4.866
F(000)	1648	1536	1536
Crystal size/ mm	0.30 x 0.25 x 0.20	0.25 x 015 x 010	0.20 x 0.10 x 0.05
θ limits, °	3.175 - 68.344	4.212 - 25.999	4.622 - 67.680
Reflections collected	46483	23038	24870
Unique obs. Reflections $[F_o > 4\sigma(F_o)]$	6505 [R_{int} = 0.0922]	6862 [R_{int} = 0.0539]	3044 [R_{int} = 0.0927]

Goodness-of-fit-on F^2	1.035	1.087	1.086
R_1 (F)[a], wR_2 (F^2) [$I > 2\sigma(I)$]	0.0480, 0.1161	0.0575, 0.1209	0.0671, 0.1476
Largest diff. peak and hole, e. Å^{-3}	1.174 and -1.086	0.714 and -0.640	0.847 and -1.571
CCDC	2326582	2326583	2326584

a) $R_1 = \Sigma||F_o|-|F_c||/\Sigma|F_o|$. [b] $wR_2 = [\Sigma w(F_o^2-F_c^2)^2/\Sigma w(F_o^2)^2]^{1/2}$ where $w = 1/[\sigma^2(F_o^2) + (aP)^2 + bP]$ where $P = (F_o^2 + F_c^2)/3$.

Crystal Packing of **3**

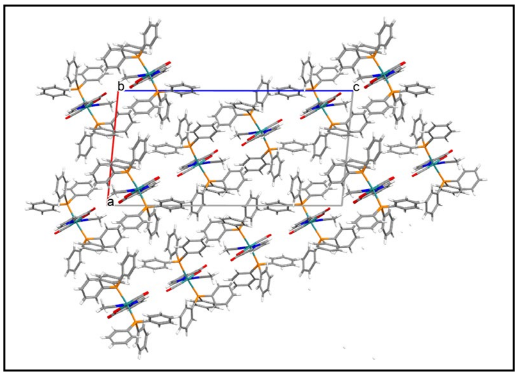

Figure S38: View down the *b* axis of the crystal packing of **3**.

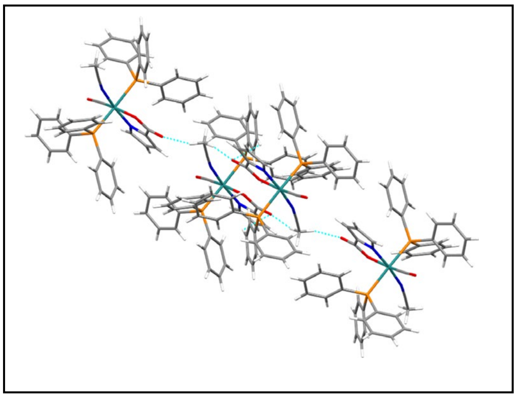

Figure S39: Arbitrary view of the packing of **3** showing intermolecular C-H…O hydrogen bonds (light-blue dots).

Crystal Packing of **4**

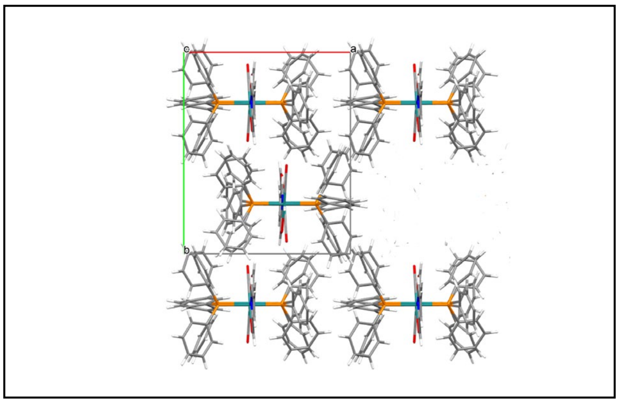

Figure S40: View down the *c* axis of the crystal packing of **4**.

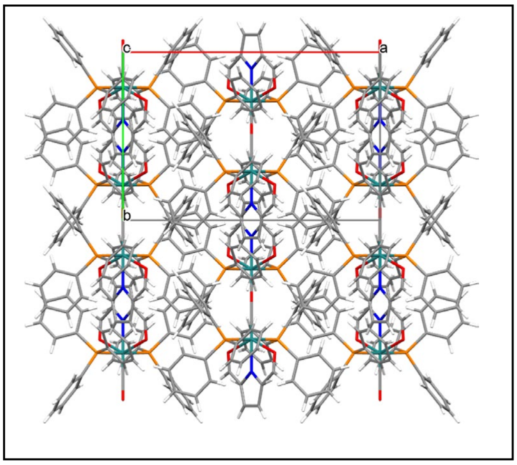

Figure S41: View down the *c* axis of the crystal packing of **5**.

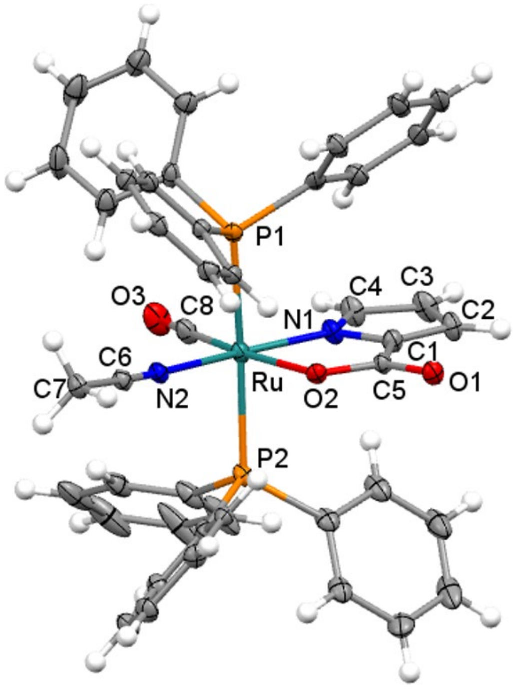

Figure S42: ORTEP drawing of **3**. Thermal ellipsoids are drawn at the 30% probability level.

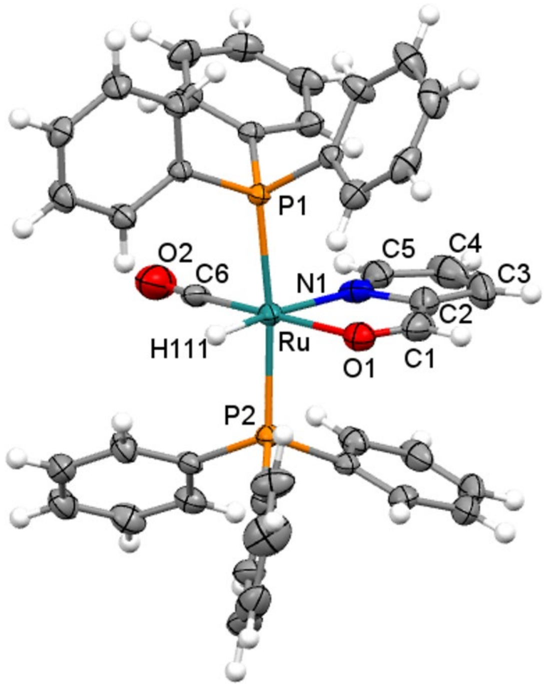

Figure S43: ORTEP drawing of **4**. Thermal ellipsoids are drawn at the 30% probability level.

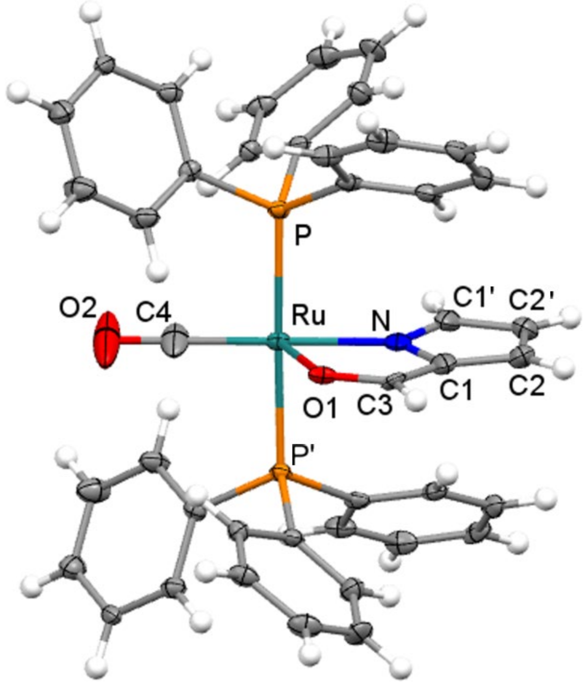

Figure S44 ORTEP drawing of **5**. Thermal ellipsoids are drawn at the 30% probability level.

Antimicrobial evaluation

Antimicrobial tests on Candida albicans

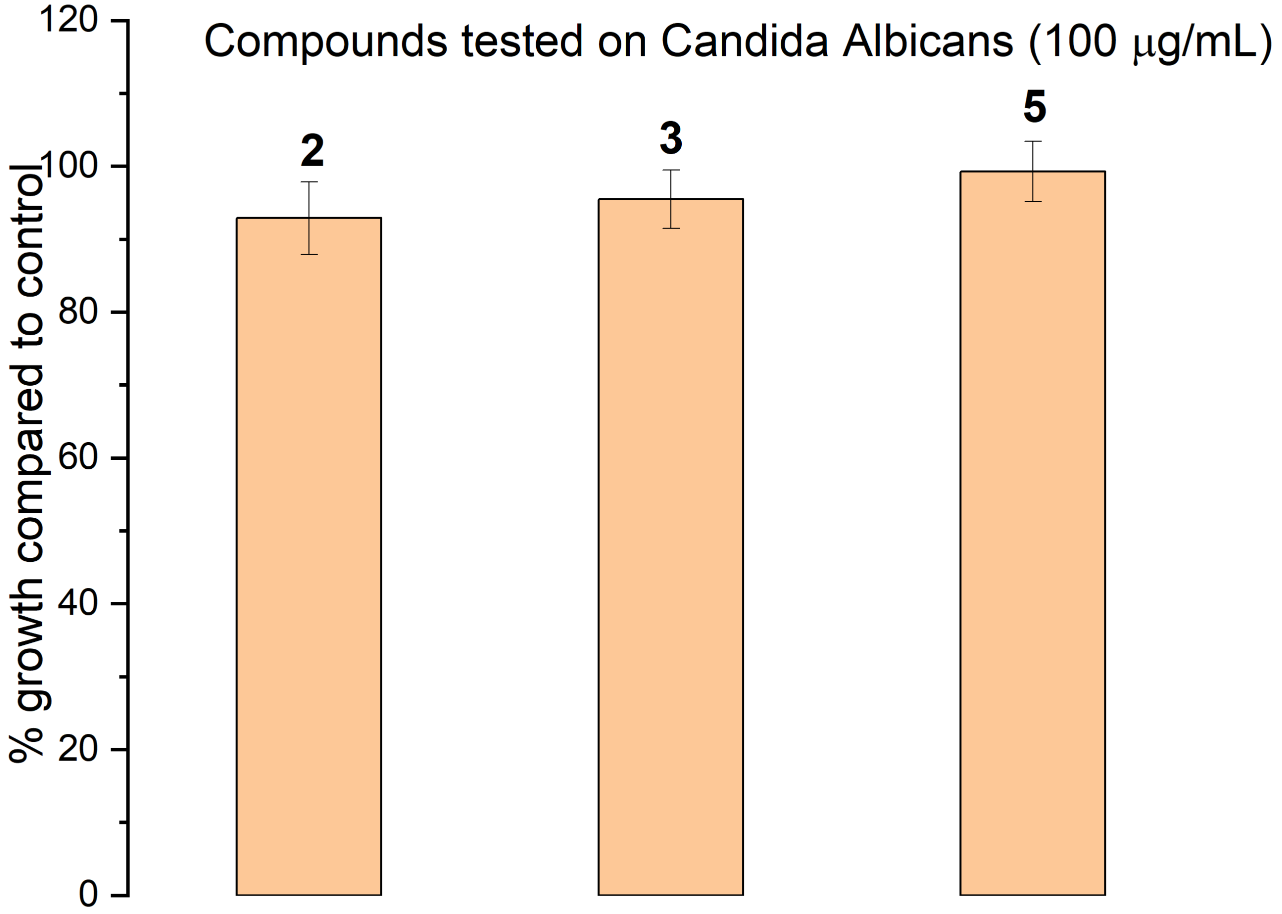

Figure S45. Percentage of the growth compared to control of Candida albicans tested with compounds **2**, **3,** and **5** by turbidity measurements at the concentration of 100 μg/mL.

Antimicrobial tests on Escherichia coli

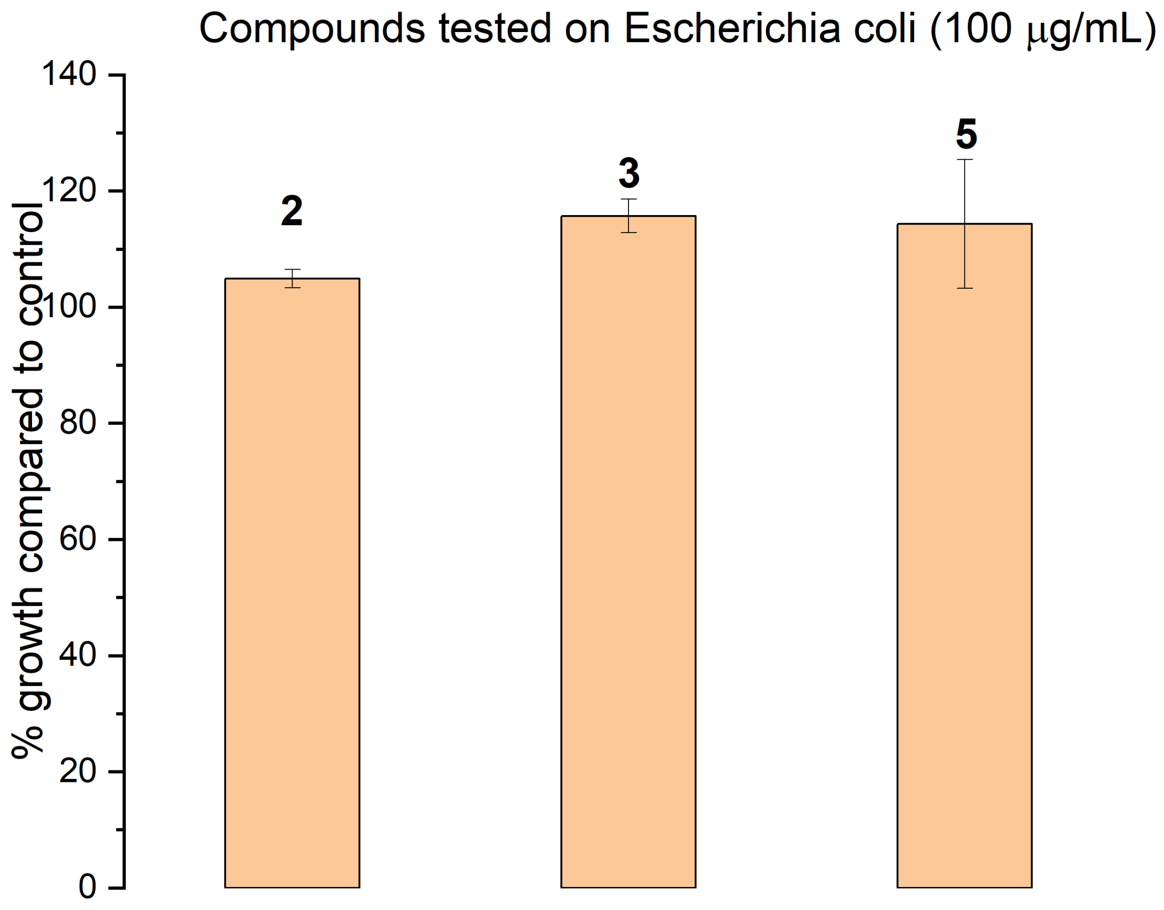

Figure S46: Percentage of the growth compared to control of Escherichia coli tested with compounds **2**, **3,** and **5** by turbidity measurements at the concentration of 100 μg/mL.

Antimicrobial tests on Staphylococcus aureus

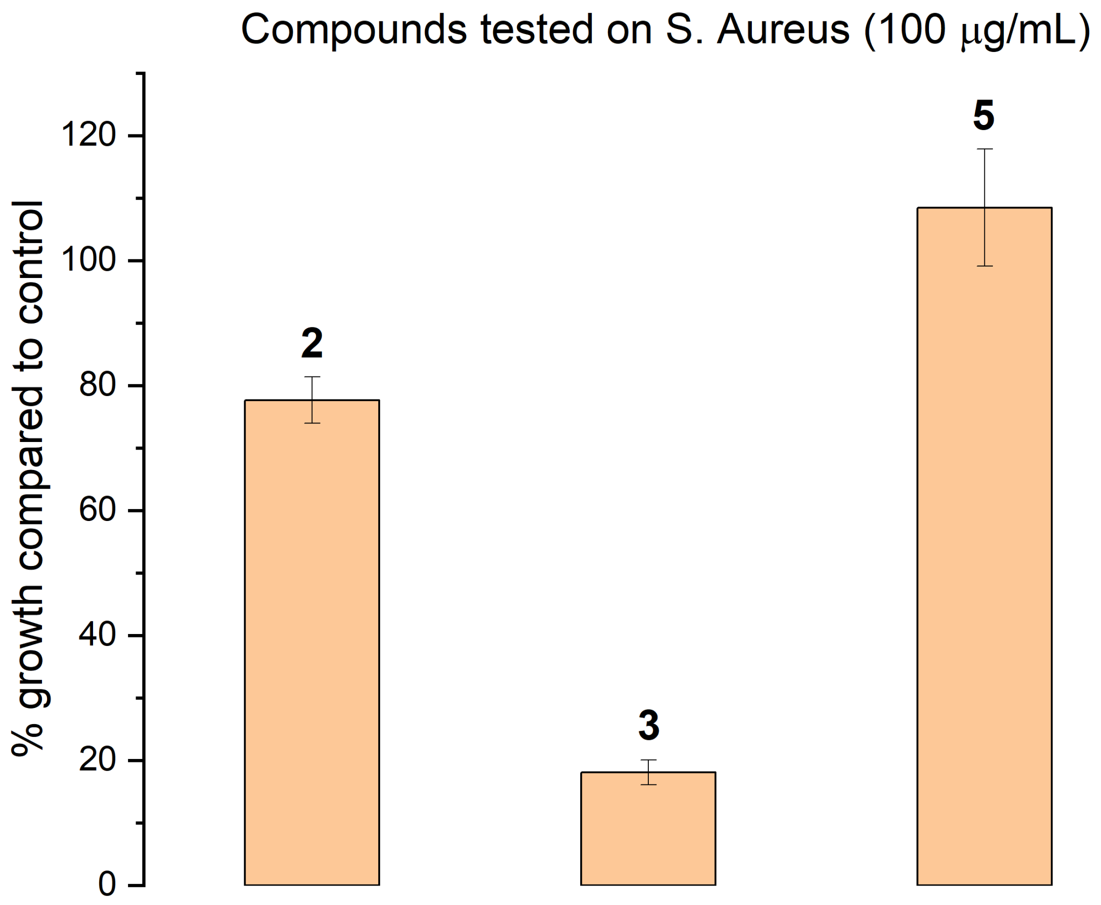

Figure S47: Percentage of the growth compared to control of Staphylococcus aureus tested with compounds **2**, **3,** and **5** by turbidity measurements at the concentration of 100 μg/mL.

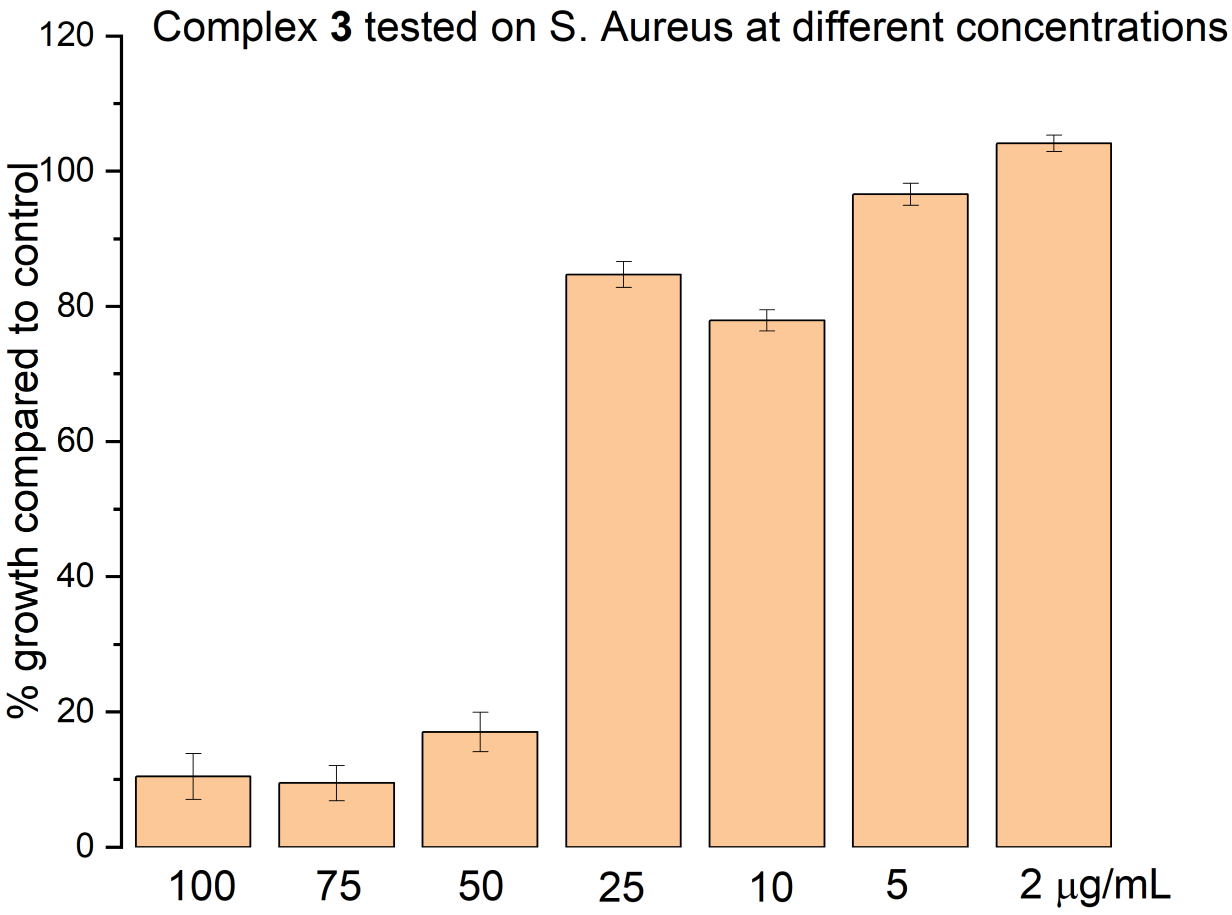

Figure S48: Percentage of the growth compared to control of Staphylococcus aureus tested with compound **3** by turbidity measurements at the concentrations of 100, 75, 50, 25, 10, 5 and 2 μg/mL.

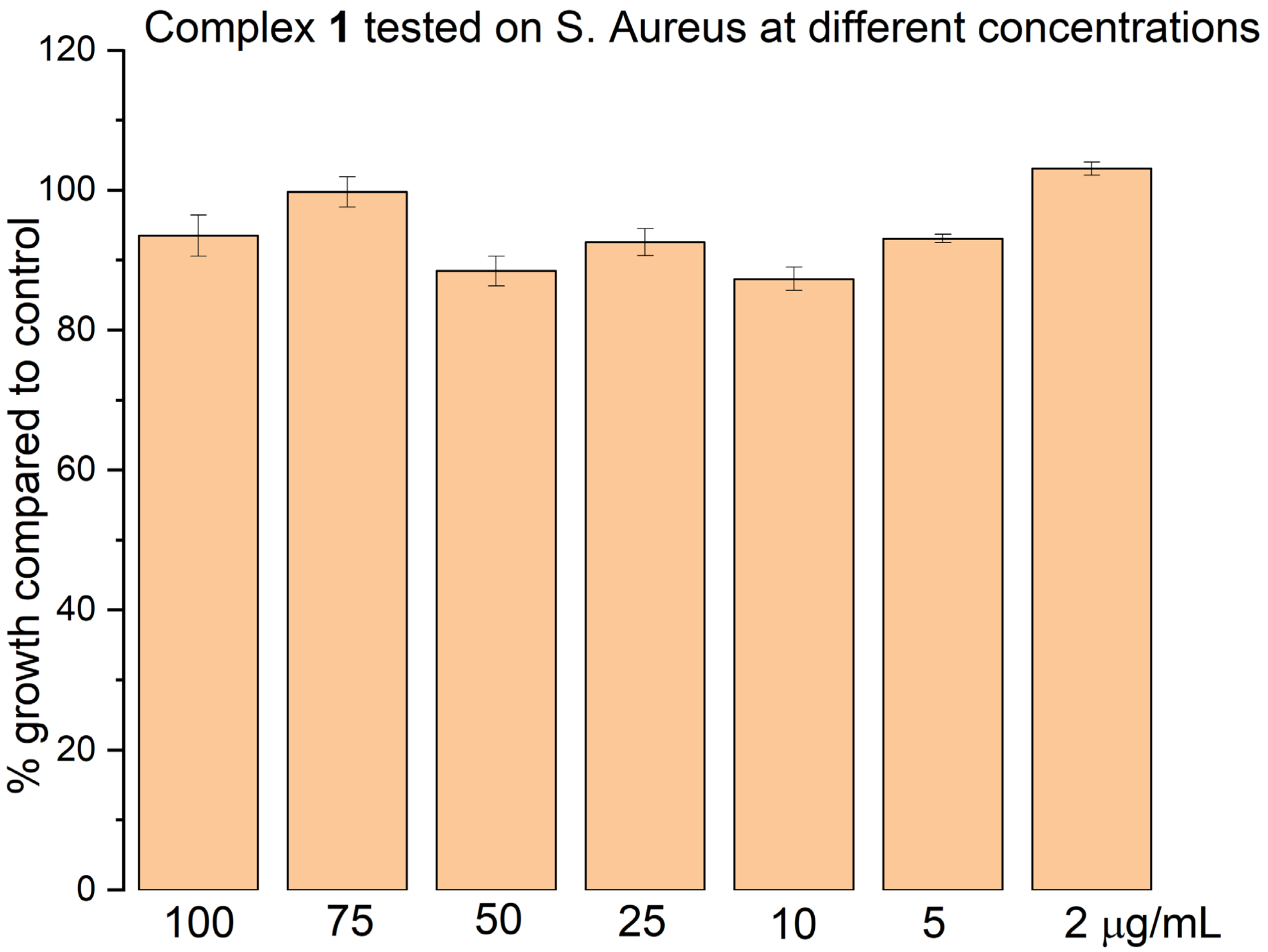

Figure S49: Percentage of the growth compared to control of Staphylococcus aureus tested with compound **1** by turbidity measurements at the concentrations of 100, 75, 50, 25, 10, 5 and 2 μg/mL.

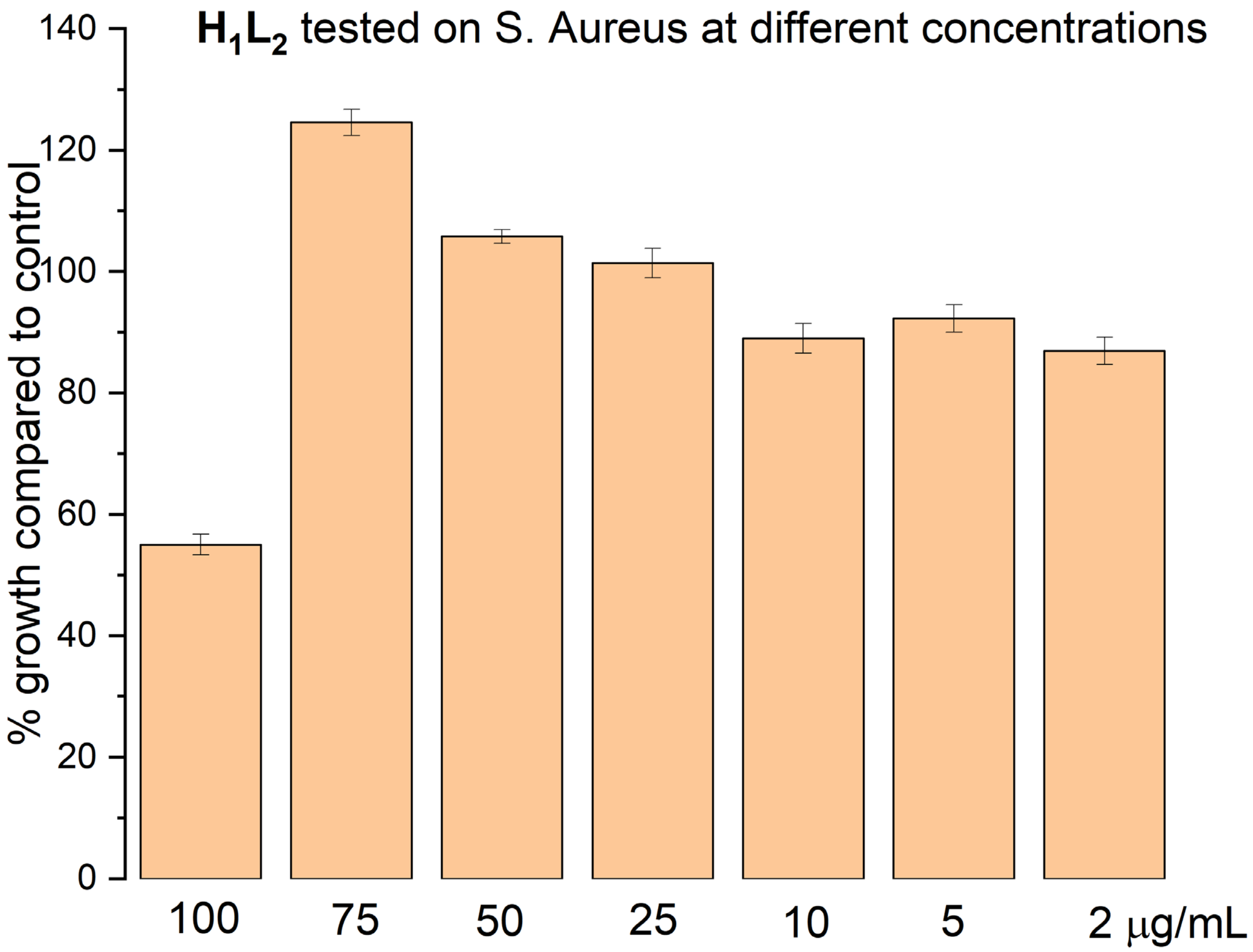

Figure S50: Percentage of the growth compared to control of Staphylococcus aureus tested with compound **H_1L_2** by turbidity measurements at the concentrations of 100, 75, 50, 25, 10, 5 and 2 μg/mL.
